# Systematic engineering of pentose phosphate pathway improves *Escherichia coli* succinate production

**DOI:** 10.1186/s13068-016-0675-y

**Published:** 2016-12-01

**Authors:** Zaigao Tan, Jing Chen, Xueli Zhang

**Affiliations:** 1 Tianjin Institute of Industrial Biotechnology, Chinese Academy of Sciences, 32 XiQiDao, Tianjin Airport Economic Park, Tianjin, 300308 China; 2 Key Laboratory of Systems Microbial Biotechnology, Chinese Academy of Sciences, Tianjin, 300308 China

**Keywords:** *Escherichia coli*, Pentose phosphate pathway (PPP), Succinate, Reducing equivalent, Ribosome binding site library (RBSL), Multivariate modular metabolic engineering (MMME)

## Abstract

**Background:**

Succinate biosynthesis of *Escherichia coli* is reducing equivalent-dependent and the EMP pathway serves as the primary reducing equivalent source under anaerobic condition. Compared with EMP, pentose phosphate pathway (PPP) is reducing equivalent-conserving but suffers from low efficacy. In this study, the ribosome binding site library and modified multivariate modular metabolic engineering (MMME) approaches are employed to overcome the low efficacy of PPP and thus increase succinate production.

**Results:**

Altering expression levels of different PPP enzymes have distinct effects on succinate production. Specifically, increased expression of five enzymes, i.e., Zwf, Pgl, Gnd, Tkt, and Tal, contributes to increased succinate production, while the increased expression of two enzymes, i.e., Rpe and Rpi, significantly decreases succinate production. Modular engineering strategy is employed to decompose PPP into three modules according to position and function. Engineering of Zwf/Pgl/Gnd and Tkt/Tal modules effectively increases succinate yield and production, while engineering of Rpe/Rpi module decreases. Imbalance of enzymatic reactions in PPP is alleviated using MMME approach. Finally, combinational utilization of engineered PPP and SthA transhydrogenase enables succinate yield up to 1.61 mol/mol glucose, which is 94% of theoretical maximum yield (1.71 mol/mol) and also the highest succinate yield in minimal medium to our knowledge.

**Conclusions:**

In summary, we systematically engineered the PPP for improving the supply of reducing equivalents and thus succinate production. Besides succinate, these PPP engineering strategies and conclusions can also be applicable to the production of other reducing equivalent-dependent biorenewables.

**Electronic supplementary material:**

The online version of this article (doi:10.1186/s13068-016-0675-y) contains supplementary material, which is available to authorized users.

## Background

Succinate, which has been identified as one of the 12 most valuable bio-bulk chemicals, has been widely used in agricultural, food, pharmaceutical, and biodegradable plastics fields, and has a potential market of $15 billion/year [1]. Construction of microbial catalysts for succinate production is a promising alternative to current petroleum-based production technics. A variety of microbes have been engineered for succinate production [2–4], among which *Escherichia coli* is recognized as an excellent biocatalyst due to its rapid growth, easy genetic manipulation, and well-known metabolism [2]. Several well-performing engineered *E. coli* strains and various fermentation technics have been developed for succinate production [3, 5–8]. Among these technics, the use of minimal medium and one-step anaerobic fermentation technology has attracted increasing attentions due to lower costs of raw materials, energy, and downstream purification [9, 10]. For instance, Jantama et al. developed a high-succinate-producing strain *E. coli* KJ073 through rational design along with metabolic evolution: using minimal AM1 10% (wt/v) glucose medium, KJ073 produced 668 mM succinate with the yield of 1.2 mol/mol glucose in simple fermentation vessels [9]. In our prior study, we also obtained a well-performing *E. coli* HX024 strain, which produces 813 mM succinate with a yield of 1.36 mol/mol glucose using AM1 12% glucose medium [8].

Despite the successes, succinate yields of these strains under minimal medium are still relatively low. Anaerobically, Embden–Meyerhof–Parnas (EMP) pathway is the predominant source of reducing equivalent: only 2 mol NADH was produced from glycolysis of 1 mol glucose, which is only enough for 1 mol succinate synthesis via the reductive TCA cycle [2]. Using EMP as the sole source of reducing equivalent, the theoretical succinate yield of 1 mol/mol glucose is only 58% of the maximal yield of 1.71 mol/mol [2]. To this end, recruitment of other reducing equivalent-conserving pathways is expected to further improve succinate production.

**Fig. 1:**
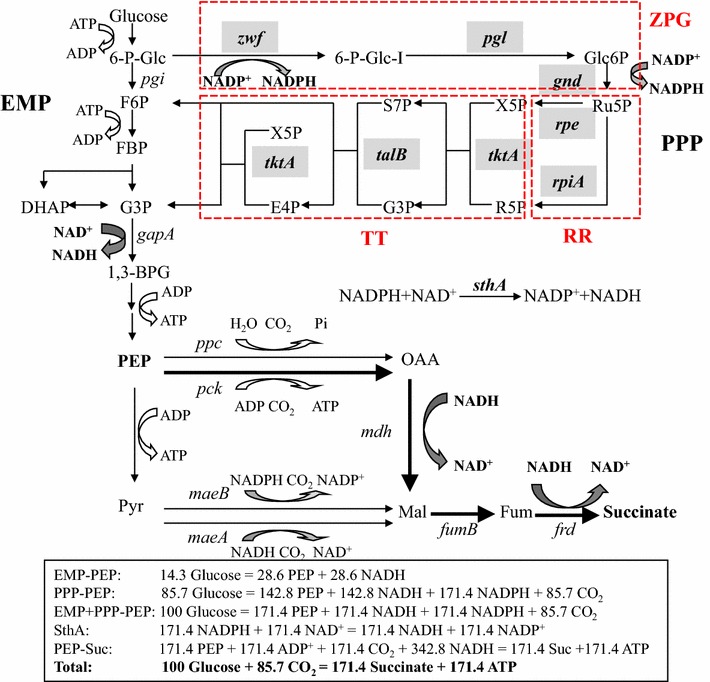
Production of succinate anaerobically in *E. coli*. Through EMP pathway, 14.3 mol glucose produces 28.6 mol PEP and 28.6 mol NADH (catalyzed by *gapA*). Through PPP, 85.7 mol glucose produces 142.8 mol PEP, 171.4 mol NADPH (catalyzed by *zwf* and *gnd*), 142.8 mol NADH (catalyzed by *gapA*), and 85.7 mol CO_2_. Through combinational utilization of EMP and PPP, 100 mol glucose produces 171.4 mol PEP, 171.4 mol NADH, 171.4 mol NADPH, and 85.7 mol CO_2_. The formed 171.4 mol NADPH can be converted into 171.4 mol NADH by increased expression of SthA. In Suc-T110, phosphoenolpyruvate carboxykinase (*pck*) is the predominant PEP carboxylase and most of PEP flux goes into the reductive TCA cycle (*mdh*-*fumB*-*frd*) for succinate biosynthesis [25]. Finally, 100 mol glucose coupled with 85.7 mol CO_2_ can produce 171.4 mol succinate, by which it achieves the theoretical maximum yield of 1.71 mol/mol glucose. EMP, Embden–Meyerhof–Parnas (glycolysis) pathway; PPP, pentose phosphate pathway; 6-P-Glc, glucose-6-phosphate; 6-P-Fru, fructose-6-phosphate; FBP, fructose-1,6-bisphosphate; 1,6-2P-Fru, 1,6-bisphosphate fructose; G3P, glyceraldehyde-3-phosphate; DHAP, dihydroxyacetone phosphate; 1,3-BPG, 1,3-bisphosphoglycerate; PEP, phosphoenolpyruvate; Pyr, pyruvate; OAA, oxaloacetate; 6-P-Glc-I, 6-phosphoglucono-δ-lactone; Glc6P, 6-phosphogluconate; Ru5P, ribulose-5-phosphate; R5P, ribose-5-phosphate; X5P, xylulose-5-phosphate; S7P, sedoheptulose-7-phosphate; E4P, erythrose-4-phosphate; F6P, fructose-6-phosphate; Mal, malate; Fum, fumarate; *pgi*, phosphoglucose isomerase; *zwf*, glucose-6-phosphate dehydrogenase; *pgl*, 6-phosphogluconolactonase; *gnd*, 6-phosphogluconate dehydrogenase; *rpiA*, ribose-5-phosphate isomerase; *rpe*, ribulose-5-phosphate-3-epimerase; *tktA*, transketolase; *talB*, transaldolase; *gapA*, glyceraldehyde 3-phosphate dehydrogenase; *ppc*, phosphoenolpyruvate carboxylase; *pck*, phosphoenolpyruvate carboxykinase; *maeA*, NAD^+^-dependent malic enzyme; *maeB*, NADP^+^-dependent malic enzyme; *mdh*, malate dehydrogenase; *fumB*, fumarase; *frd*, fumarate reductase; *sthA*, soluble pyridine nucleotide transhydrogenase; ADP, adenosine diphosphate; ATP, adenosine triphosphate; NAD^+^, oxidized nicotinamide adenine dinucleotide; NADH, reduced nicotinamide adenine dinucleotide; NADP^+^, oxidized nicotinamide adenine dinucleotide phosphate; NADPH, reduced nicotinamide adenine dinucleotide phosphate

Compared with EMP, pentose phosphate pathway (PPP) is reducing equivalent-conserving: degrading 1 mol glucose provides 3.67 mol NAD(P)H (Fig. 1). *Escherichia coli* PPP consists of seven enzymes, i.e., glucose-6-phosphate dehydrogenase (Zwf), 6-phosphogluconolactonase (Pgl), 6-phosphogluconate dehydrogenase (Gnd), ribose-5-phosphate isomerase (Rpi), ribulose-5-phosphate 3-epimerase (Rpe), transketolase (Tkt), and transaldolase (Tal) (Fig. 1). Increasing carbon flux through PPP has been experimentally demonstrated to increase reducing equivalent supply and production of products [11–13]. For instance, increased expression of Zwf increased ε-caprolactone production by 39% in *E. coli* harboring cyclohexanone monooxygenase gene [11]. Introduction of metabolite-resistant Gnd mutant of PPP into *Corynebacterium glutamicum* AHP-3 increased l-lysine production by 15% [14]. Activating TktA in our prior study increased succinate yield from 1.12 to 1.26 mol/mol glucose [8]. Despite these successes, this central carbon metabolic pathway has not been systematically engineered. Some questions still remain largely unexplored. First, besides Zwf, Gnd, and Tkt, can any other enzymes impact the efficiency of PPP and serve as metabolic engineering targets for PPP? Second, plasmid overexpression was usually used to increase enzyme expression. However, there might not be a linear correlation between the expression level of each enzyme and PPP efficiency. What is the optimal expression level of each specific enzyme to obtain an efficient PPP? Finally, given that excessive expression of a single enzyme might lead to metabolic imbalance that may compromise cellular growth and pathway efficiency, systematic engineering of PPP is desirable [15–17].

Multivariate modular metabolic engineering (MMME) approach has been proposed to balance the metabolic flux among pathway modules. Modules with varied expression levels (low, medium, or high) are combined to search the best combination for product biosynthesis, and this approach has achieved successes in the production of bio-products, e.g., taxadiene, fatty acids, and isoprene [15, 18, 19]. In order to obtain modules with low, medium, and high expression levels, combining promoters with varied strength and plasmids with different copy numbers becomes the commonly used strategy [15, 18, 19]. Although being useful, this strategy might suffer from metabolic burden from excessive formation of transcriptional mRNA and plasmid maintenance [3, 20, 21]. In contrast, altering the expression at transcriptional level opens a promising alternative route. Since ribosome binding site (RBS) is responsible for recruitment of ribosome for the initiation of protein translation, changing the sequence of RBS is reasonable to affect the efficiency of translational initiation and thus protein synthesis [22]. Therefore, the construction of RBS library (RBSL) via introduction of degenerate nucleotides at RBS region [23, 24] will provide a variety of enzyme or module candidates with varied expression levels available for MMME.

In this study, we systematically engineered the entire PPP via RBSL and MMME, using succinate as an example target product. *E. coli* Suc-T110 (∆*pflB* ∆*ldhA* ∆ptsI Ppck*-galP pck*) [25] was selected as the starting strain for its relatively low succinate production (titer ~280 mM, yield ~1.12 mol/mol glucose) [8], and no reducing equivalent-conserving pathway has been activated within. RBSL approach was initially employed to obtain PPP enzymes with varied expression levels. Results revealed that, besides Zwf, Gnd, and Tkt, increased expression of other PPP enzymes, e.g., Pgl and Tal, also improved succinate production within a certain range, while the increased expression of Rpe or Rpi instead compromised succinate production. Then, PPP was decomposed into three modules (ZPG, RR, and TT), the MMME approach was used to search the best combination of both intra- and inter-modules. Finally, combinational utilization of engineered PPP and SthA transhydrogenase enables succinate yield of *E. coli* Suc-P02 up to 1.61 mol/mol glucose, which increases by 44% relative to starting Suc-T110 and also the highest yield (94% of theoretical maximum yield) in minimal medium to our knowledge.

## Results

### Engineering effects of individual PPP enzymes on succinate production

We first measured the activities of all PPP enzymes within Suc-T110 under anaerobic condition. All of these PPP enzymes were found to have relatively low expression levels, with activities ranging from 0.05 to 0.71 U/mg (Additional file 1: Table S1). Given that transcriptional regulation is the widely used strategy for *E. coli* to regulate gene expression [26], we proposed that the low activities of PPP enzymes anaerobically are probably due to transcriptional repression from oxygen-sensitive transcriptional regulators, e.g., FNR [27]. Consistent with our hypothesis, some FNR-binding sites are found to be present at the upstream transcriptional regulatory region of PPP genes, e.g., *zwf* and *gnd* [28].

**Fig. 2:**
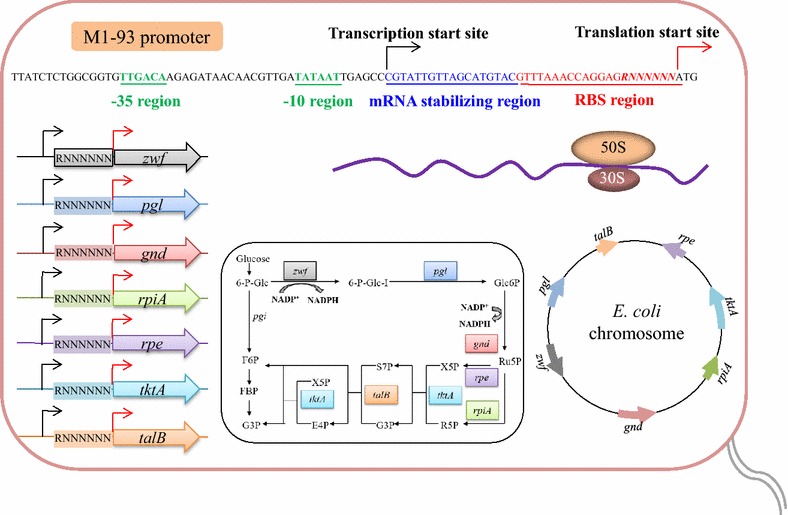
Schematic of the construction of ribosome binding site library (RBSL) of PPP enzymes. The seven PPP enzymes locate dispersedly among *E. coli* MG1655 chromosome. A constitutive promoter M1-93 was initially used to replace native promoter of PPP enzymes. Next, seven degenerate nucleotides (*RNNNNNN*) were introduced into the RBS region of M1-93 promoter before ATG start codon to obtain the RBSL

**Fig. 3:**
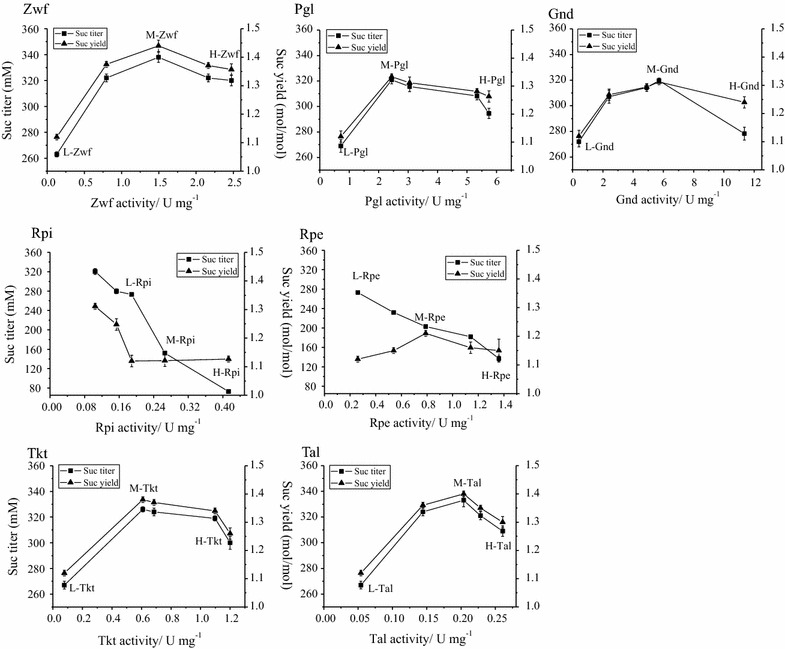
Increased expression of PPP enzymes for succinate production. Relationship between the expression level (enzymatic activity) of each PPP enzyme and succinate production. *Square symbol* represents succinate *titer* and *triangle symbol* represents succinate yield. Values are the average of three biological replicates with *error bars* indicating one standard deviation. L, low expression level; M, medium expression level; H, high expression level

With the goal of relieving the transcriptional repression and thus increasing expression levels of PPP enzymes, we next employed the RBSL approach to replace the native promoter of all PPP enzymes by the constitutive M1-93 artificial promoter [29, 30] with varied RBS sequence (Fig. 2; Additional file 2: Table S2). Seven degenerate nucleotides (*RNNNNNN*) were introduced to the RBS region (before the ATG start codon) of PPP genes (Fig. 2). After promoter replacement, the expression levels of PPP enzymes increased significantly (Additional file 3: Table S3). For Zwf, its highest expression level reached 2.47 U/mg, which is 19-fold of native expression level (0.13 U/mg) in Suc-T110. For other Pgl, Gnd, Rpi, Rpe, Tkt, and Tal enzymes, the highest expression levels increased by 8-, 27-, 2-, 5-, 17-, and 5-fold at most relative to the respective native expression levels in Suc-T110 (Fig. 3; Additional file 3: Table S3). Moreover, given that altering sequences of RBS will affect the efficiency of translational initiation and protein synthesis [22], we also obtained a series of PPP enzymes with varied expression levels (Fig. 3; Additional file 3: Table S3).

Subsequently, we investigated the correlation between enzymatic expression levels and succinate production in these PPP engineered strains (Fig. 3). For Zwf, Pgl, Gnd, Tkt, and Tal enzymes, it was observed that there is a positive correlation between the expression levels and succinate production when the expression level is within a certain range (Fig. 3), while excessive expression leads to decreased succinate production but still higher than the starting strain Suc-T110 (Fig. 3). Taking Zwf enzyme for instance, the maximum succinate production was obtained when Zwf expression level increased to the medium level of 1.50 U/mg (M-Zwf), which enabled a 29% increase in succinate titer to 338 mM and also a 29% increase in succinate yield to 1.44 mol/mol glucose relative to Suc-T110 with a low level of Zwf (L-Zwf, 0.13 U/mg) (*P* < 0.05). However, when Zwf expression level further elevated to as high as 2.47 U/mg (H-Zwf), succinate titer decreased by 5% to 320 mM and succinate yield also decreased by 5% to 1.36 mol/mol compared with M-Zwf (*P* < 0.05) (Fig. 3; Additional file 3: Table S3). Similar trend is also applicable to Pgl, Gnd, Tkt, and Tal enzymes and the optimal expression levels of Pgl, Gnd, Tkt, and Tal for succinate production were identified at the medium levels of 3.05, 5.71, 0.68, and 0.20 U/mg, respectively (Fig. 3; Additional file 3: Table S3).

In contrast, this trend is not applicable to Rpi or Rpe enzymes, which locate at the metabolic branch of PPP (Fig. 1). In contrast, a negative relationship was observed between the Rpe or Rpi expression level and succinate production (Fig. 3; Additional file 3: Table S3). For Rpi, when its expression level increased from 0.19 U/mg (L-Rpi) by 42% to 0.27 U/mg (M-Rpi), although there was no substantial change in succinate yield, succinate titer significantly decreased by 44% to 152 mM (*P* < 0.05). This suppression effect becomes more obvious when Rpi expression level further elevated to 0.41 U/mg (H-Rpi), which decreased succinate titer by 73% to 73 mM (*P* < 0.05) (Fig. 3). We observed that the compromised succinate production is associated with decreased biomass, which declined by 64% to 0.56 g/l in M-Rpi and by 85% to 0.23 g/l in H-Rpi (Additional file 3: Table S3). Similarly, when Rpe expression level increased from 0.27 U/mg (L-Rpe) by twofold to 0.79 U/mg (M-Rpe), succinate titer decreased by 25% to 203 mM, while the biomass decreased by 37% to 0.98 g/l, and 1.36 U/mg of Rpe expression level (H-Rpe) further decreased succinate titer and biomass by 50% to 137 mM and by 54% to 0.71 g/l, respectively (*P* < 0.05) (Fig. 3; Additional file 3: Table S3).

### Modular engineering of PPP for increasing succinate production

Traditionally, PPP is divided into the oxidative part (including Zwf, Pgl, and Gnd) and the non-oxidative part (including Rpi, Rpe, Tkt, and Tal) [31]. However, given that the reactions in the non-oxidative part are distinct (isomerization vs. aldehyde/ketone transfer), it is more reasonable to further divide the non-oxidative part into the Rpe/Rpi and Tkt/Tal parts. Therefore, the entire PPP is partitioned into three modules: (I) Zwf/Pgl/Gnd (ZPG) module, which converts glucose-6-phosphate (G6P) into ribulose-5-phosphate (Ru5P) and CO_2_, and generates NADPH; (II) Rpe/Rpi (RR) module, which converts Ru5P into xylulose-5-phosphate (X5P) and ribose-5-phosphate (R5P); and (III) Tkt/Tal (TT) module, which converts X5P and R5P into fructose-6-phosphate (F6P) and glyceraldehyde-3-phosphate (G3P) that enter the glycolytic pathway for further metabolism (Fig. 1).

**Fig. 4:**
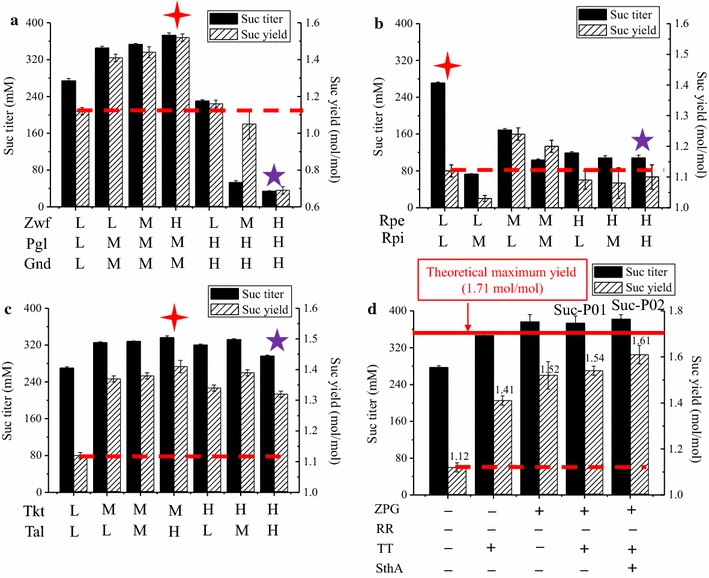
Modular engineering of PPP for succinate production. Effect of engineering **a** ZPG module, **b** RR module, and **c** TT module on succinate production. **d** Combinational utilization of optimized ZPG module (H-Zwf/M-Pgl/M-Gnd, labeled by 4-point star in **a**), optimized TT module (M-Tkt/H-Tal, labeled by 4-point star in **c**), and increased expression of SthA for succinate production. The final succinate yield in Suc-P02 is up to 1.61 mol/mol glucose, which increased by 44% over the starting strain Suc-T110 (~1.12 mol/mol). Values are the average of three biological replicates with *error bars* indicating one standard deviation. The 4-point star labels the best-performing succinate-producing strain during individual module engineering; the 5-point star indicates the strain with excessive expression of single module accompanied with significantly decreased succinate production. L, low expression level; M, medium expression level; H, high expression level

To optimize each module for succinate production, the multivariate engineering approach was proposed and employed [15, 18, 19]. Enzymes with varied expression levels, i.e., low (L), medium (M), and high (H), were chosen to perform the intra-module combinations. During the optimization of ZPG module, M-Pgl and M-Gnd were found to have an additive effect in increasing succinate production. Specifically, M-Pgl and M-Gnd individually led to a 17% increase in succinate yield. However, M-Pgl/M-Gnd combination enabled engineered *E. coli* strain to produce 345 mM succinate with the yield of 1.41 mol/mol glucose, which led to a 26% increase in the yield over Suc-T110 (1.12 mol/mol glucose) (*P* < 0.05) (Fig. 4a; Additional file 4: Table S4). Combinational utilization of the increased expression of Zwf and M-Pgl/M-Gnd further increased succinate production (Fig. 4a; Additional file 4: Table S4). For instance, H-Zwf/M-Pgl/M-Gnd strain produced 376 mM succinate with the yield of 1.52 mol/mol, which is 36% higher than that produced by Suc-T110 (*P* < 0.05). However, excessive expression of Pgl and Gnd (H-Pgl/H-Gnd) decreased succinate production: engineered strain H-Zwf/H-Pgl/H-Gnd only produced 34 mM succinate with a yield of 0.69 mol/mol glucose (Fig. 4a; Additional file 4: Table S4).

During engineering of RR module, we found that, although increased expression levels of RR module will cause a slight increase in succinate yield, succinate titer significantly decreased (Fig. 4b; Additional file 4: Table S4). For example, M-Rpe/M-Rpi enabled *E. coli* to produce only 104 mM succinate, which is far lower than the starting strain Suc-T110 (L-Rpe/l-Rpi, ~280 mM succinate) (*P* < 0.05).

Individual increased expression of Tkt or Tal led to increased succinate production (Fig. 3). In addition, combinational utilization of Tkt and Tal enzymes further increased succinate production. For instance, succinate titer and yield of M-Tkt/H-Tal strain were 336 mM and 1.41 mol/mol glucose, respectively, which exceeded those when using H-Tal alone (1.30 mol/mol) by more than 9% (*P* < 0.05) (Fig. 4c; Additional file 4: Table S4). However, excessive expression level of Tkt and Tal enzymes was found to compromise succinate production. Specifically, H-Tkt/H-Tal strain only produced 296 mM succinate with the yield of 1.32 mol/mol glucose, which decreased by 6 and 6% compared with M-Tkt/T-Tal (336 mM; 1.41 mol/mol, *P* < 0.05) (Fig. 4c; Additional file 4: Table S4).

Since engineering of each PPP module increased succinate production, it is reasonable to expect that combinational utilization of PPP modules will further improve succinate production. Given that increased expression of RR module does not contribute to succinate production, we then attempted to combine the ZPG and TT modules. Given that the H-Zwf/M-Pgl/M-Gnd and M-Tkt/H-Tal manipulations are identified to be the most effective during individual engineering of ZPG module and TT module (Fig. 4a, c, labeled by 4-point star). Combinational utilization of the two manipulations together resulted in the engineered *E. coli* strain Suc-P01 (H-Zwf/M-Pgl/M-Gnd/M-Tkt/H-Tal). Results showed that Suc-P01 produced 373 mM succinate with the yield of 1.54 mol/mol glucose (Fig. 4d), which is higher than that obtained when using M-Tkt/H-Tal alone (1.41 mol/mol) (*P* < 0.05). It is to be noted that the difference between individual utilization of H-Zwf/M-Pgl/M-Gnd and Suc-P01 is marginal (Fig. 4d).

### Alleviating metabolic bottlenecks through MMME approach

In this study, by systematic engineering of PPP at the levels of enzyme and module, we have successfully obtained *E. coli* strains with highly active PPP and well-performing succinate production. However, some questions remain to be solved, e.g., excessive expression of individual enzyme or module compromised pathway performance and succinate production. Specifically, succinate titers of H-ZPG, H-RR, and H-TT are only 34, 106, and 296 mM, respectively (Fig. 4a–c, labeled by 5-point star). We proposed that these poor performances are presumably due to metabolic bottlenecks caused by the mismatch of modules. To this end, the MMME approach was employed again to alleviate this potential metabolic imbalance.

**Fig. 5:**
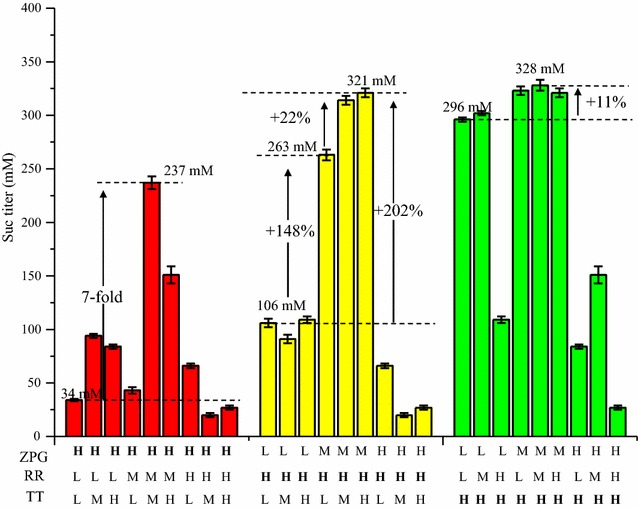
Alleviating metabolic imbalance via the MMME approach. Succinate titers of the starting strains with H-ZPG, H-RR, and H-TT are only 34, 106, and 296 mM, respectively. For H-ZPG, the M-RR/M-TT leads to a sevenfold increase of succinate to 237 mM. For H-RR, the M-ZPG intervention increases its titer by 148% to 263 mM, and following H-TT modification, it further increases by 22% to 321 mM. For H-TT, M-ZPG/M-RR finally increased succinate titer by 11% to 328 mM. Values are the average of three biological replicates with *error bars* indicating one standard deviation. L, low expression level; M, medium expression level; H, high expression level; MMME, modified multivariate modular metabolic engineering

We found that the poor performance of H-ZPG can be advanced by the activation of downstream RR and TT modules. Specifically, strain H-ZPG/M-RR/M-TT produced 237 mM succinate, which increased sevenfold than H-ZPG (34 mM) (Fig. 5; Additional file 5: Table S5). Given that Ru5P is the catalytic product of ZPG, H-ZPG might cause the accumulation of Ru5P intermediate, tuning of the expression of downstream RR and TT modules from low to medium level (M-RR/M-TT) contributed to alleviating this metabolic bottleneck and thus recovered succinate production. It is to be noted that succinate-producing capability of the resulting H-ZPG/M-RR/M-TT strain is still not outstanding: succinate titer (237 mM) was only about 85% of the parental strain Suc-T110 (~280 mM), indicating that metabolic imbalance caused by H-ZPG was only partially alleviated.

H-RR might cause metabolic bottleneck of Ru5P shortage; here, we revealed that it can be alleviated by increasing the expression of upstream ZPG and TT modules to medium level (M-ZPG/M(H)-TT), and the former impacts predominantly due to providing more Ru5P intermediate for H-RR. For example, M-ZPG/H-RR produced 263 mM succinate, which exceeded that of H-RR (106 mM) by 148% (*P* < 0.05) (Fig. 5; Additional file 5: Table S5). Interestingly, under this condition, activation of downstream TT module can further increase succinate production. Specifically, M-ZPG/H-RR/H-TT produced 321 mM succinate, which even exceeded that of M-ZPG/H-RR by 22% (*P* < 0.05).

For H-TT, activation of RR module compromised succinate production (Fig. 5). Instead, activation of ZPG module increased succinate production. For instance, M-ZPG/M-RR/H-TT produced 328 mM succinate, which increased by 11% compared with H-TT (296 mM) (*P* < 0.05) (Fig. 5; Additional file 5: Table S5). Activation of ZPG module might increase the supply of key intermediates (e.g., X5P, R5P, E4P, and S7P) for H-TT metabolism, which relieves the metabolic bottleneck of H-TT.

All of these results demonstrated the effectiveness of MMME approach for solving metabolic imbalance from mismatch of PPP modules. However, it should be pointed out that succinate productions of all these MMME strains are still lower than that of Suc-P01 (titer = 373 mM, yield = 1.54 mol/mol glucose), which underlines the importance of fine-tuning expression level of each module for activation of PPP.

### Combinational utilization of engineered PPP and SthA transhydrogenase for succinate production

SthA is a soluble transhydrogenase responsible for converting NADPH into NADH in *E. coli* [8, 32]. Our prior research showed that the increased expression of SthA in *E. coli* increased succinate production due to improving supply of NADH for succinate biosynthesis [8]. Since the key enzymes of both malate dehydrogenase (MDH) and fumarate dehydrogenase (FRD) in the succinate biosynthesis pathway are NADH dependent, efficient conversion of NADPH into NADH after engineering of PPP might contribute to further improvement of succinate production. To this end, the expression level of SthA transhydrogenase was increased by replacing the native promoter of *sthA* with a strong constitutive promoter RBS3 in Suc-P01 strain, the best PPP engineered strain obtained in this study, resulting in Suc-P02 strain (H-Zwf/M-Pgl/M-Gnd/M-Tkt/M-Tal/RBS3-SthA). Compared with Suc-P01, expression of SthA in Suc-P02 increased onefold from 0.03 to 0.06 U/mg. This increased SthA expression enabled Suc-P02 to produce 382 mM succinate with the yield of 1.61 mol/mol glucose (Fig. 4d; Additional file 4: Table S4), which exceeded those of Suc-P01 (1.54 mol/mol) and the starting strain Suc-T110 (1.12 mol/mol) by 5 and 44% (*P* < 0.05) respectively, and also 94% of the theoretical maximum yield (1.71 mol/mol). To our knowledge, this is the highest succinate yield of *E. coli* in minimal medium so far.

## Discussion

In this study, the entire PPP was systematically investigated for its capability to supply reducing equivalents for succinate production. Under anaerobic condition, the low expression levels of PPP enzymes have been successfully advanced via RBSL approach, which indicates that the low efficacy of PPP anaerobically is at least partially due to expressional repression. Moreover, by introduction of degenerate nucleotides in the RBS region, a variety of engineered strains with different expression levels of PPP enzymes have been obtained. Further analysis revealed that the increased expression of Zwf, Pgl, Gnd, Tkt, and Tal enzymes contributes to succinate production, which provides more candidates for engineering of PPP besides the widely used Zwf and Gnd [11, 13]. Although being useful at low levels, excessive expression of the five enzymes compromised succinate production, presumably due to the accumulation of inhibitory metabolic intermediates [33]. In contrast, the increased expression of Rpe or Rpi enzyme, which locate at the metabolic branch of PPP, decreased succinate production. We proposed that it might be attributed to the imbalance of R5P and X5P in the engineered strains. For downstream TT module, it requires 2 molecules of X5P (**X5P** + R5P → S7P + G3P; **X5P** + E4P → F6P + G3P), while it requires only 1 molecule of R5P (X5P + **R5P** → S7P + G3P) for entire aldehyde/ketone transfer reactions. Therefore, the theoretical optimal mol ratio between X5P and R5P should be 2:1. Increased expression levels of individual Rpi or Rpe will break the balance of X5P and R5P, which leads to the actual mol ratio deviate the optimal 2:1. In contrast to Rpe, increased expression of Rpi is severer for that TT module needs more X5P (2 molecules) relative to R5P (1 molecule) to accomplish the entire transfer reactions.

Modular engineering strategy was employed to decompose PPP into three modules. Among PPP modules, optimization of the ZPG module exhibited the most effective increase in succinate production, improving succinate yield from 1.12 by 36% to 1.52 mol/mol glucose. We deemed that the effectiveness of ZPG optimization might be due to the two NADPH-generating enzymes, i.e., Zwf and Gnd, both of which locate at this module. Besides ZPG, optimization of TT module also contributed to succinate production, increasing succinate yield from 1.12 by 26% to 1.41 mol/mol glucose. TT module locates at the downstream of PPP and connects with the EMP pathway. Increased expression of TT module will accelerate the metabolic rate of PPP metabolic products, such as G3P and F6P, to enter the EMP downstream pathway for further metabolism. For RR module, compared with individual increased expression of Rpe or Rpi enzyme, simultaneous increased expression of Rpe/Rpi has not yet been proven the perfect solution for solving R5P and X5P imbalance for that it might cause new metabolic bottleneck of Ru5P intermediate shortage, leading to decreased biomass and succinate production (Additional file 5: Table S5). This explanation is confirmed by the experimental result that the activation of upstream ZPG module based on H-Rpi/H-Rpe significantly increased biomass from 0.63 by 76% to 1.11 g/l and increased succinate titer from 106 mM by 148% to 263 mM (Additional file 5: Table S5). Maintaining the native expression levels of Rpe and Rpi enzymes seems to be the best solution for the optimization of RR module. Consequently, combinational utilization of the most effective ZPG and TT modules further increased succinate yield by 38% to 1.54 mol/mol. It should be noted that after systematic optimization of PPP, the native expression level of SthA is not as high to convert all of the produced NADPH into NADH for succinate biosynthesis and increased expression of SthA based on ZPG + TT further was found to further increase succinate yield by 44% to 1.61 mol/mol glucose. In the future, fine-tuning expression of SthA based on the activation of PPP might lead to further succinate yield improvement.

Excessive expression of single PPP enzyme or module will cause potential metabolic imbalance (e.g., intermediate shortage or accumulation) and thus decrease succinate production. We deem that the changes in intracellular concentration of PPP intermediates will cause two detrimental effects. First, it compromises PPP activity and efficacy. Second, cellular growth will be retarded as some PPP intermediates have been reported to be associated with cell growth. For instance, R5P, E4P, and S7P are required for biosynthesis in *E. coli* cells, and 6-phosphoglucono-δ-lactone (6-P-Glc-I) is deemed the cytotoxic chemical which can react with endogenous nucleophiles [34–36]. In this study, MMME approach has proven its effectiveness in alleviating these potential metabolic imbalances, which recovers PPP efficiency, biomass, and succinate production.

Sekar et al. reported that the overexpression of NADPH-generating Zwf and Gnd can activate PPP regardless of down-regulation of the EMP pathway, and the EMP pathway must be down-regulated to enhance the glycolytic flux through PPP [37, 38]. Here we found that besides Zwf and Gnd, activation of other indirect NADPH-generating enzymes of PPP, i.e., Pgl, Tkt, and Tal, also contributes to the improvement of PPP efficacy. Furthermore, although down-regulation of EMP flux (e.g., deletion of *pgi* or *pfkA*) is the commonly used strategy for channeling flux to PPP, our results indicated that fine-tuning expression of PPP enzymes is indispensable for obtaining highly active PPP. Combinational utilization of the down-regulation of EMP and fine-tuning expression of PPP enzymes in future will have an additive role in further increasing the production of reducing equivalents and related products.

In this study, a series of *E. coli* strains with increased succinate production have been obtained. For instance, engineered strain Suc-P02 has the succinate yield of 1.61 mol/mol glucose, which is 94% of the maximal theoretical yield (1.71 mol/mol) and also the highest in minimal medium. It is to be noted that even though we deleted *ldhA* and *pflB* competitive pathways, a small quantity of acetate remains in fermentation broth of Suc-P02. Deletion of other possible acetate formation pathways, such as *ackA*, *pta*, or *poxB*, might contribute to further succinate yield improvement. Overall, this work highlights the importance of systematic engineering of PPP for the supply of reducing equivalents for succinate production, strategies of which can be applied to the production of other reducing equivalent-dependent microbial biorenewables.

## Conclusions

The pentose phosphate pathway of *E. coli* was systematically engineered for improving reducing equivalent supply and succinate production. Increased expression of each of Zwf, Pgl, Gnd, Tkt, and Tal enzymes contributed to increased succinate production, while the increased expression of either Rpe or Rpi significantly decreased succinate production. Through combined engineering of Zwf/Pgl/Gnd, Tkt/Tal, and SthA modules, succinate yield reached 1.61 mol/mol glucose, which was 94% of theoretical maximum yield (1.71 mol/mol) and also the highest succinate yield in minimal medium to our knowledge.

## Methods

### Strains, medium, and growth conditions

*Escherichia coli* strains used in this study can be found in the additional materials (Additional file 6: Table S6). During construction process, the strains were cultured at 30 or 37 °C in Luria broth (10 g/l Difco tryptone, 5 g/l Difco yeast extract and 10 g/L NaCl). If necessary, ampicillin, kanamycin, or chloramphenicol was added to the medium at a final concentration of 100, 40, or 17 μg/ml, respectively [25].

### Genetic methods

Primers used in this study are listed in Additional file 7: Table S7. For the construction of ribosome binding site libraries (RBSL) [23, 25], a two-step recombination approach was performed [39]. Taking the construction of zwf-RBSL for instance, the *zwf*-*cat*-*sacB* genetic cassette was amplified from pXZ-CS plasmid [25] with primer set zwf-cat-sacB-up/down and inserted before the ATG start codon of *zwf* gene. Then, primer set zwf-P-up/zwf-RBSL-down and *E. coli* M1-93 genomic DNA [40] were used to PCR amplify the zwf-RBSL DNA fragment for the second recombination step. Seven degenerate nucleotides (*RNNNNNN*) will be introduced into the RBS region before the ATG start codon of *zwf* gene (see Fig. 2). After PCR verification using zwf-EX-up/down, ten right colonies were randomly picked from each RBSL for further measuring the Zwf activities. Four representative strains having varied Zwf activities were selected for fermentation and the regulatory elements before the ATG start codon of *zwf* gene in these four engineered strains were sequenced and are listed in Additional file 2: Table S2.

### Fermentation

Fresh colonies were picked from New Brunswick Scientific (NBS) mineral salt plates containing 20 g/l glucose, inoculated into 250-ml flasks containing 100 ml mineral salt medium with 50 g/l glucose, and grown at 37 °C and 100 rpm for 12 h. The seed cultures were then inoculated into a 500-ml fermentation vessel containing 250 ml mineral salt medium. Potassium bicarbonate was added to the fermentation medium with a final concentration of 100 mM. The pH was maintained at 7.0 by automatic addition of a base containing 2.4 M sodium carbonate and 1.2 M sodium hydroxide [25].

### Enzyme assay

All of the kinetic parameters of *E. coli *PPP enzymes can be found in Additional file 8: Table S8. Crude extracts were prepared from cells harvested during the mid-log phase (60 h) of fermentation. Collected *E. coli* cells were firstly washed with 50 mM Tris buffer (pH 7.0) twice and then suspended in the same buffer with 1× protease inhibitor (Roche, Switzerland) to an OD_550_ of 10 for sonication treatment. After centrifugation at 12,000×*g* and 4 °C for 20 min, the supernatant (crude extract) was transferred to a new tube. Bio-Rad Protein Assay Kit (Bio-Rad, USA) was used to measure protein concentration of the crude extract. One unit (U) of enzyme activity represents the amount of enzyme catalyzing the conversion of 1 µmol of substrate per min into specific products. The extinction coefficient of NADPH and NADH at 340 nm was 6.22/cm mM.

#### Zwf (EC 1.1.1.49) activity

Zwf activity was determined as previously described with minor modifications [41, 42]. Each 1 ml reaction mixture contains 10 mM MgCl_2_, 1 mM DTT, 0.5 mM NADP^+^, 2 mM glucose-6-phosphate, and crude extract in 100 mM Tris buffer (pH 7.5). One unit of enzyme activity was defined as 1 µmol NADPH formed/min mg/protein.

#### Pgl (EC 3.1.1.31) activity

Two additional enzymes, Zwf and Gnd, were added to the reaction mixture for Pgl activity assay. The 6-phosphoglucono-δ-lactone formed by Zwf was catalyzed by Pgl to form 6-phosphogluconate, which was converted subsequently by Gnd to form Ru5P, accompanied with NADPH formation. Each 1 ml reaction mixture contains 2 mM MgCl_2_, 0.5 mM 6-phosphate-glucose, 1 mM NADP^+^, and 10 U Zwf in 25 mM HEPES buffer (pH 7.1). After incubation at dark for 8 min, 1.5 U Gnd and crude extract were added to the mixture [43]. One unit of enzyme activity was defined as 1 µmol NADPH formed/min mg/protein.

#### Gnd (EC 1.1.1.44) activity

Gnd activity was determined as previously described with minor modifications [44]. Each 1 ml reaction mixture contains 10 mM MgCl_2_, 1 mM DTT, 0.5 mM NADP^+^, 2 mM 6-phosphate-gluconate, and crude extract in 100 mM Tris buffer (pH 7.5). One unit of enzyme activity was defined as 1 µmol NADPH formed/min mg/protein.

#### Rpe (EC 5.1.3.1) activity

Rpe activity was determined as previously described with minor modifications [45]. Each 1 ml reaction mixture contains 0.24 mM MgCl_2_, 0.01 mM TPP, 0.25 mM NADH, 3 U 3-phosphate-glycerol dehydrogenase, 10 U triosephosphate isomerase, 0.5 mM d-ribose-5-phosphate, 0.5 mM d-ribulose-5-phosphate, 1 U transketolase, and crude extract in 50 mM Tris buffer (pH 7.5). One unit of enzyme activity was defined as 1 µmol NADH decreased/min mg/protein.

#### Rpi (EC 5.3.1.6) activity

Rpi activity was determined as previously described with minor modifications [45]. Each 1 ml reaction mixture contains 0.24 mM MgCl_2_, 0.01 mM TPP, 0.25 mM NADH, 3 U 3-phosphate-glycerol dehydrogenase, 10 U triosephosphate isomerase, 0.5 mM d-xylulose-5-phosphate, 0.5 mM d-ribulose-5-phosphate, 1 U transketolase, and crude extract in 50 mM Tris buffer (pH 7.5). One unit of enzyme activity was defined as 1 µmol NADH decreased/min mg/protein.

#### Tkt (EC 2.2.1.1) activity

Tkt activity was determined as previously described with minor modifications [45]. Each 1 ml reaction mixture contains 0.24 mM MgCl_2_, 0.01 mM TPP, 0.25 mM NADH, 3 U 3-phosphate-glycerol dehydrogenase, 10 U triosephosphate isomerase, 0.5 mM d-ribose-5-phosphate, 0.5 mM d-xylulose-5-phosphate, and crude extract in 50 mM Tris buffer (pH 7.5). One unit of enzyme activity was defined as 1 µmol NADH decreased/min mg/protein.

#### Tal (EC 2.2.1.2) activity

Tal activity was determined as previously described with minor modifications [31]. HEPES buffer instead of Tris buffer was used to wash and suspend cells. Each 1 ml reaction mixture contains 0.24 mM MgCl_2_, 0.5 mM NADP^+^, 0.5 mM d-sedoheptulose-7-phosphate, 0.5 mM glyceraldehyde-3-phosphate, 10 U 6-phosphate-glucose isomerase, 3 U 6-phosphate-glucose dehydrogenase, and crude extract in 100 mM HEPES buffer (pH 8.5). One unit of enzyme activity was defined as 1 µmol NADPH formed/min mg/protein.

#### SthA (EC 1.6.1.1) activity

 SthA activity was determined as previously described [46]. The crude extract after sonication was firstly centrifuged at 12,000×*g* and 4 °C for 5 min and then further centrifuged at 50,000×*g* and 4 °C for 60 min. The obtained supernatant was transferred for use. Each 1 ml reaction mixture contains 2 mM MgCl_2_, 2 mM NADPH, 3 U 3-acetyl pyridine adenine dinucleotide (APAD^+^), and crude extract in 50 mM Tris buffer (pH 7.5). One unit of enzyme activity was defined as increased 1 μmol APADH/min mg/protein with an extinction coefficient of 2.9/cm mM at 400 nm.

### Analysis

The dry weight of cells was calculated by measuring the optical density value at 550 nm (OD_550_). Organic acids and residual glucose in the fermentation broth were measured by high-performance liquid chromatography [47]. The product titers were normalized by arithmetically factoring out the volume of base solution that was added to the fermenters for pH control as follows: product titer = (real product titer) × (starting volume + added base solution volume)/(starting volume) [8]. Two-tailed *t* test was employed to analyze the statistical significance of all the data, and a *P* value <0.05 is deemed as statistically significant.

## Additional files

**Additional file 1.** Activities of PPP enzymes in Suc-T110 during succinate production.

**Additional file 2.** Sequences of regulatory elements in engineering of PPP.

**Additional file 3.** Production of succinate by engineering individual PPP enzymes .

**Additional file 4.** Modular engineering PPP for succinate production.

**Additional file 5.** Solving metabolic burdens through multivariate modular engineering.

**Additional file 6.** Genotypes of all strains used in this study.

**Additional file 7.** Primers used in this study.

**Additional file 8.** Kinetic parameters of E. coli PPP enzymes.

## Abbreviations

EMP: Embden–Meyerhof–Parnas pathway
PPP: pentose phosphate pathway
RBSL: ribosome binding site library
MMME: multivariate modular metabolic engineering
Zwf: glucose-6-phosphate dehydrogenase
Pgl: 6-phosphogluconolactonase
Gnd: 6-phosphogluconate dehydrogenase
Rpi: ribose-5-phosphate isomerase
Rpe: ribulose-5-phosphate-3-epimerase
Tkt: transketolase
Tal: transaldolase
ZPG: Zwf/Pgl/Gnd
RR: Rpe/Rpi
TT: Tkt/Tal
G3P: glyceraldehyde-3-phosphate
6-P-Glc-I: 6-phosphoglucono-δ-lactone
Glc6P: 6-phosphogluconate
Ru5P: ribulose-5-phosphate
R5P: ribose-5-phosphate
X5P: xylulose-5-phosphate
S7P: sedoheptulose-7-phosphate
E4P: erythrose-4-phosphate
F6P: fructose-6-phosphate
Suc: succinate
L: low
M: medium
H: high

## References

[1] McKinlay JB, Vieille C, Zeikus JG (2007). Prospects for a bio-based succinate industry. Appl Microbiol Biotechnol.

[2] Cheng KK, Wang GY, Zeng J, Zhang JA (2013). Improved succinate production by metabolic engineering. Biomed Res Int.

[3] Jarboe LR, Zhang X, Wang X, Moore JC, Shanmugam KT, Ingram LO (2010). Metabolic engineering for production of biorenewable fuels and chemicals: contributions of synthetic biology. J Biomed Biotechnol.

[4] Liu P, Jarboe LR (2012). Metabolic engineering of biocatalysts for carboxylic acids production. Comput Struct Biotechnol J.

[5] Chatterjee R, Millard CS, Champion K, Clark DP, Donnelly MI (2001). Mutation of the *ptsG* gene results in increased production of succinate in fermentation of glucose by *Escherichia coli*. Appl Environ Microbiol.

[6] Vemuri GN, Eiteman MA, Altman E (2002). Effects of growth mode and pyruvate carboxylase on succinic acid production by metabolically engineered strains of *Escherichia coli*. Appl Environ Microbiol.

[7] Zhang X, Jantama K, Moore JC, Jarboe LR, Shanmugam KT, Ingram LO (2009). Metabolic evolution of energy-conserving pathways for succinate production in *Escherichia coli*. Proc Natl Acad Sci USA.

[8] Zhu X, Tan Z, Xu H, Chen J, Tang J, Zhang X (2014). Metabolic evolution of two reducing equivalent-conserving pathways for high-yield succinate production in *Escherichia coli*. Metab Eng.

[9] Jantama K, Haupt MJ, Svoronos SA, Zhang X, Moore JC, Shanmugam KT, Ingram LO (2008). Combining metabolic engineering and metabolic evolution to develop nonrecombinant strains of *Escherichia coli* C that produce succinate and malate. Biotechnol Bioeng.

[10] Liu P, Zhu X, Tan Z, Zhang X, Ma Y (2016). Construction of *Escherichia coli* cell factories for production of organic acids and alcohols. Adv Biochem Eng Biotechnol.

[11] Lee WH, Park JB, Park K, Kim MD, Seo JH (2007). Enhanced production of epsilon-caprolactone by overexpression of NADPH-regenerating glucose 6-phosphate dehydrogenase in recombinant *Escherichia coli* harboring cyclohexanone monooxygenase gene. Appl Microbiol Biotechnol.

[12] Wang Y, San KY, Bennett GN (2013). Improvement of NADPH bioavailability in *Escherichia coli* through the use of phosphofructokinase deficient strains. Appl Microbiol Biotechnol.

[13] Zhao J, Li Q, Sun T, Zhu X, Xu H, Tang J, Zhang X, Ma Y (2013). Engineering central metabolic modules of *Escherichia coli* for improving β-carotene production. Metab Eng.

[14] Ohnishi J, Katahira R, Mitsuhashi S, Kakita S, Ikeda M (2005). A novel gnd mutation leading to increased l-lysine production in *Corynebacterium glutamicum*. FEMS Microbiol Lett.

[15] Ajikumar PK, Xiao WH, Tyo KE, Wang Y, Simeon F, Leonard E, Mucha O, Phon TH, Pfeifer B, Stephanopoulos G (2010). Isoprenoid pathway optimization for Taxol precursor overproduction in *Escherichia coli*. Science.

[16] Du J, Yuan Y, Si T, Lian J, Zhao H (2012). Customized optimization of metabolic pathways by combinatorial transcriptional engineering. Nucleic Acids Res.

[17] Pitera DJ, Paddon CJ, Newman JD, Keasling JD (2007). Balancing a heterologous mevalonate pathway for improved isoprenoid production in *Escherichia coli*. Metab Eng.

[18] Lv X, Gu J, Wang F, Xie W, Liu M, Ye L, Yu H (2016). Combinatorial pathway optimization in *Escherichia coli* by directed co-evolution of rate-limiting enzymes and modular pathway engineering. Biotechnol Bioeng.

[19] Xu P, Gu Q, Wang W, Wong L, Bower AG, Collins CH, Koffas MA (2013). Modular optimization of multi-gene pathways for fatty acids production in *E. coli*. Nat Commun.

[20] Togna AP, Shuler ML, Wilson DB (1993). Effects of plasmid copy number and runaway plasmid replication on overproduction and excretion of beta-lactamase from *Escherichia coli*. Biotechnol Prog.

[21] Jones KL, Kim SW, Keasling JD (2000). Low-copy plasmids can perform as well as or better than high-copy plasmids for metabolic engineering of bacteria. Metab Eng.

[22] Chen HY, Bjerknes M, Kumar R, Jay E (1994). Determination of the optimal aligned spacing between the Shine-Dalgarno sequence and the translation initiation codon of *Escherichia coli* mRNAs. Nucleic Acids Res.

[23] Chen J, Zhu X, Tan Z, Xu H, Tang J, Xiao D, Zhang X (2013). Activating C-dicarboxylate transporters DcuB and DcuC for improving succinate production. Appl Microbiol Biotechnol.

[24] Salis HM, Mirsky EA, Voigt CA (2009). Automated design of synthetic ribosome binding sites to control protein expression. Nat Biotechnol.

[25] Tan Z, Zhu X, Chen J, Li Q, Zhang X (2013). Activating phosphoenolpyruvate carboxylase and phosphoenolpyruvate carboxykinase in combination for improvement of succinate production. Appl Environ Microbiol.

[26] Balleza E, Lopez-Bojorquez LN, Martinez-Antonio A, Resendis-Antonio O, Lozada-Chavez I, Balderas-Martinez YI, Encarnacion S, Collado-Vides J (2009). Regulation by transcription factors in bacteria: beyond description. FEMS Microbiol Rev.

[27] Kang YS, Weber KD, Yu Q, Kiley PJ, Blattner FR (2005). Genome-wide expression analysis indicates that FNR of *Escherichia coli* K-12 regulates a large number of genes of unknown function. J Bacteriol.

[28] Kumar R, Shimizu K (2011). Transcriptional regulation of main metabolic pathways of *cyoA*, *cydB*, *fnr*, and *fur* gene knockout *Escherichia coli* in C-limited and N-limited aerobic continuous cultures. Microb Cell Fact.

[29] Tang J, Zhu X, Lu J, Liu P, Xu H, Tan Z, Zhang X (2013). Recruiting alternative glucose utilization pathways for improving succinate production. Appl Microbiol Biotechnol.

[30] Tan Z, Yoon JM, Nielsen DR, Shanks JV, Jarboe LR (2016). Membrane engineering via trans unsaturated fatty acids production improves *Escherichia coli* robustness and production of biorenewables. Metab Eng.

[31] Sprenger GA (1995). Genetics of pentose-phosphate pathway enzymes of *Escherichia coli* K-12. Arch Microbiol.

[32] Sauer U, Canonaco F, Heri S, Perrenoud A, Fischer E (2004). The soluble and membrane-bound transhydrogenases UdhA and PntAB have divergent functions in NADPH metabolism of *Escherichia coli*. J Biol Chem.

[33] Dahl RH, Zhang F, Alonso-Gutierrez J, Baidoo E, Batth TS, Redding-Johanson AM, Petzold CJ, Mukhopadhyay A, Lee TS, Adams PD, Keasling JD (2013). Engineering dynamic pathway regulation using stress-response promoters. Nat Biotechnol.

[34] Miclet E, Stoven V, Michels PAM, Opperdoes FR, Lallemand JY, Duffieux F (2001). NMR spectroscopic analysis of the first two steps of the pentose-phosphate pathway elucidates the role of 6-phosphogluconolactonase. J Biol Chem.

[35] Moat AG, Foster JW, Spector MP. Introduction to microbial physiology. Microbial Physiology. 4th ed. 1995. p. 1-26.

[36] Tarighi S, Wei Q, Camara M, Williams P, Fletcher MP, Kajander T, Cornelis P (2008). The PA4204 gene encodes a periplasmic gluconolactonase (PpgL) which is important for fitness of *Pseudomonas aeruginosa*. Microbiology.

[37] Seol E, Sekar BS, Raj SM, Park S (2016). Co-production of hydrogen and ethanol from glucose by modification of glycolytic pathways in *Escherichia coli* - from Embden-Meyerhof-Parnas pathway to pentose phosphate pathway. Biotechnol J.

[38] Sekar BS, Seol E, Raj SM, Park S (2016). Co-production of hydrogen and ethanol by *pfkA*-deficient *Escherichia coli* with activated pentose-phosphate pathway: reduction of pyruvate accumulation. Biotechnol Biofuels.

[39] Shi AQ, Zhu XN, Lu J, Zhang XL, Ma YH (2013). Activating transhydrogenase and NAD kinase in combination for improving isobutanol production. Metab Eng.

[40] Lu J, Tang J, Liu Y, Zhu X, Zhang T, Zhang X (2012). Combinatorial modulation of *galP* and *glk* gene expression for improved alternative glucose utilization. Appl Microbiol Biotechnol.

[41] Kabir MM, Shimizu K (2003). Gene expression patterns for metabolic pathway in *pgi* knockout *Escherichia coli* with and without *phb* genes based on RT-PCR. J Biotechnol.

[42] Lamed R, Zeikus JG (1980). Glucose fermentation pathway of *Thermoanaerobium brockii*. J Bacteriol.

[43] Stanford DR, Whitney ML, Hurto RL, Eisaman DM, Shen WC, Hopper AK (2004). Division of labor among the yeast sol proteins implicated in tRNA nuclear export and carbohydrate metabolism. Genetics.

[44] Padilla L, Kramer R, Stephanopoulos G, Agosin E (2004). Overproduction of trehalose: heterologous expression of *Escherichia coli* trehalose-6-phosphate synthase and trehalose-6-phosphate phosphatase in *Corynebacterium glutamicum*. Appl Environ Microbiol.

[45] Sobota JM, Imlay JA (2011). Iron enzyme ribulose-5-phosphate 3-epimerase in *Escherichia coli* is rapidly damaged by hydrogen peroxide but can be protected by manganese. Proc Natl Acad Sci USA.

[46] Chin JW, Khankal R, Monroe CA, Maranas CD, Cirino PC (2009). Analysis of NADPH supply during xylitol production by engineered *Escherichia coli*. Biotechnol Bioeng.

[47] Zhang X, Jantama K, Shanmugam KT, Ingram LO (2009). Reengineering *Escherichia coli* for succinate production in mineral salts medium. Appl Environ Microbiol.

