# Supplementary material for: Systematic engineering of pentose phosphate pathway improves Escherichia coli succinate production
Source: Biotechnol Biofuels. 2016 Dec 1;9:262. doi: 10.1186/s13068-016-0675-y (PMC5134279; doi:10.1186/s13068-016-0675-y)
Supplement: Supplementary file 1 — Additional file 1. Activities of PPP enzymes in Suc-T110 during succinate production. [file 13068_2016_675_MOESM1_ESM.doc]

**Additional Table S1. Activities of PPP enzymes in Suc-T110 during succinate production**

| Name | Enzyme activity (µmol/mg min) |
| --- | --- |
| Zwf (EC 1.1.1.49) | 0.13±0.01 |
| Pgl (EC 3.1.1.31) | 0.71±0.06 |
| Gnd (EC 1.1.1.44) | 0.42±0.03 |
| Rpi (EC 5.3.1.6) | 0.19±0.03 |
| Rpe (EC 5.1.3.1) | 0.27±0.04 |
| Tkt (EC 2.2.1.1) | 0.07±0.02 |
| Tal (EC 2.2.1.2) | 0.054±0.006 |
