# Supplementary material for: Systematic engineering of pentose phosphate pathway improves Escherichia coli succinate production
Source: Biotechnol Biofuels. 2016 Dec 1;9:262. doi: 10.1186/s13068-016-0675-y (PMC5134279; doi:10.1186/s13068-016-0675-y)
Supplement: Supplementary file 2 — Additional file 2. Sequences of regulatory elements in engineering of PPP. [file 13068_2016_675_MOESM2_ESM.doc]

**Additional Table S2. Sequences of regulatory elements in engineering of PPP**

| **Name** | **Sequence** |
| --- | --- |
| *RBSL1-zwf* | TTATCTCTGGCGGTG**TTGACA**AGAGATAACAACGTTGA**TATAAT**TGAGCCCGTATTGTTAGCATGTACGTTTAAACCAGGAG*AGACAGA* |
| *RBSL2-zwf* | TTATCTCTGGCGGTG**TTGACA**AGAGATAACAACGTTGA**TATAAT**TGAGCCCGTATTGTTAGCATGTACGTTTAAACCAGGAG*AGAGCTA* |
| *RBSL3-zwf* | TTATCTCTGGCGGTG**TTGACA**AGAGATAACAACGTTGA**TATAAT**TGAGCCCGTATTGTTAGCATGTACGTTTAAACCAGGAG*AAGTAAA* |
| *RBSL4-zwf* | TTATCTCTGGCGGTG**TTGACA**AGAGATAACAACGTTGA**TATAAT**TGAGCCCGTATTGTTAGCATGTACGTTTAAACCAGGAG*ATGTATC* |
|  |  |
| *RBSL1-pgl* | TTATCTCTGGCGGTG**TTGACA**AGAGATAACAACGTTGA**TATAAT**TGAGCCCGTATTGTTAGCATGTACGTTTAAACCAGGAG*GCGGAAC* |
| *RBSL2-pgl* | TTATCTCTGGCGGTG**TTGACA**AGAGATAACAACGTTGA**TATAAT**TGAGCCCGTATTGTTAGCATGTACGTTTAAACCAGGAG*GCACCAA* |
| *RBSL3-pgl* | TTATCTCTGGCGGTG**TTGACA**AGAGATAACAACGTTGA**TATAAT**TGAGCCCGTATTGTTAGCATGTACGTTTAAACCAGGAG*ACGCCCA* |
| *RBSL4-pgl* | TTATCTCTGGCGGTG**TTGACA**AGAGATAACAACGTTGA**TATAAT**TGAGCCCGTATTGTTAGCATGTACGTTTAAACCAGGAG*AGGAGCT* |
|  |  |
| *RBSL1-gnd* | TTATCTCTGGCGGTG**TTGACA**AGAGATAACAACGTTGA**TATAAT**TGAGCCCGTATTGTTAGCATGTACGTTTAAACCAGGAG*GACCAGG* |
| *RBSL2-gnd* | TTATCTCTGGCGGTG**TTGACA**AGAGATAACAACGTTGA**TATAAT**TGAGCCCGTATTGTTAGCATGTACGTTTAAACCAGGAG*ACAACGA* |
| *RBSL3-gnd* | TTATCTCTGGCGGTG**TTGACA**AGAGATAACAACGTTGA**TATAAT**TGAGCCCGTATTGTTAGCATGTACGTTTAAACCAGGAG*GCGACTA* |
| *RBSL4-gnd* | TTATCTCTGGCGGTG**TTGACA**AGAGATAACAACGTTGA**TATAAT**TGAGCCCGTATTGTTAGCATGTACGTTTAAACCAGGAG*AAGTCAA* |
|  |  |
| *RBSL1-rpiA* | TTATCTCTGGCGGTG**TTGACA**AGAGATAACAACGTTGA**TATAAT**TGAGCCCGTATTGTTAGCATGTACGTTTAAACCAGGAG*GGAAGAG* |
| *RBSL2-rpiA* | TTATCTCTGGCGGTG**TTGACA**AGAGATAACAACGTTGA**TATAAT**TGAGCCCGTATTGTTAGCATGTACGTTTAAACCAGGAG*GAAACAC* |
| *RBSL3-rpiA* | TTATCTCTGGCGGTG**TTGACA**AGAGATAACAACGTTGA**TATAAT**TGAGCCCGTATTGTTAGCATGTACGTTTAAACCAGGAG*AAACCGC* |
| *RBSL4-rpiA* | TTATCTCTGGCGGTG**TTGACA**AGAGATAACAACGTTGA**TATAAT**TGAGCCCGTATTGTTAGCATGTACGTTTAAACCAGGAG*AGACAAG* |
|  |  |
| *RBSL1-rpe* | TTATCTCTGGCGGTG**TTGACA**AGAGATAACAACGTTGA**TATAAT**TGAGCCCGTATTGTTAGCATGTACGTTTAAACCAGGAG*GCTAAGC* |
| *RBSL2-rpe* | TTATCTCTGGCGGTG**TTGACA**AGAGATAACAACGTTGA**TATAAT**TGAGCCCGTATTGTTAGCATGTACGTTTAAACCAGGAG*AACGAAC* |
| *RBSL3-rpe* | TTATCTCTGGCGGTG**TTGACA**AGAGATAACAACGTTGA**TATAAT**TGAGCCCGTATTGTTAGCATGTACGTTTAAACCAGGAG*GACGAGT* |
| *RBSL4-rpe* | TTATCTCTGGCGGTG**TTGACA**AGAGATAACAACGTTGA**TATAAT**TGAGCCCGTATTGTTAGCATGTACGTTTAAACCAGGAG*GAGGTGC* |
|  |  |
| *RBSL1-tktA* | TTATCTCTGGCGGTG**TTGACA**AGAGATAACAACGTTGA**TATAAT**TGAGCCCGTATTGTTAGCATGTACGTTTAAACCAGGAG*AGACAAG* |
| *RBSL2-tktA* | TTATCTCTGGCGGTG**TTGACA**AGAGATAACAACGTTGA**TATAAT**TGAGCCCGTATTGTTAGCATGTACGTTTAAACCAGGAG*GTACCCT* |
| *RBSL3-tktA* | TTATCTCTGGCGGTG**TTGACA**AGAGATAACAACGTTGA**TATAAT**TGAGCCCGTATTGTTAGCATGTACGTTTAAACCAGGAG*AAAATCA* |
| *M1-37-tktA* | TTATCTCTGGCGGTG**TTGACA**AGAGATAACAACGTTGA**TATAAT**TGAGCCCGTATTGTTAGCATGTACGTTTAAACCAGGAA*ACAGCTA* |
|  |  |
| *RBSL1-talB* | TTATCTCTGGCGGTG**TTGACA**AGAGATAACAACGTTGA**TATAAT**TGAGCCCGTATTGTTAGCATGTACGTTTAAACCAGGAG*ATCTAAG* |
| *RBSL3-talB* | TTATCTCTGGCGGTG**TTGACA**AGAGATAACAACGTTGA**TATAAT**TGAGCCCGTATTGTTAGCATGTACGTTTAAACCAGGAG*AAGAGTA* |
| *RBSL3-talB* | TTATCTCTGGCGGTG**TTGACA**AGAGATAACAACGTTGA**TATAAT**TGAGCCCGTATTGTTAGCATGTACGTTTAAACCAGGAG*GTGCGAG* |
| *RBSL4-talB* | TTATCTCTGGCGGTG**TTGACA**AGAGATAACAACGTTGA**TATAAT**TGAGCCCGTATTGTTAGCATGTACGTTTAAACCAGGAG*GGCCGTA* |

The -35 (**TTGACA**) and -10 (**TATAAT**) consensus sequences are in bold, the varied RBS sequences (*RNNNNNN*) was italicized and underlined.
