# Supplementary material for: Systematic engineering of pentose phosphate pathway improves Escherichia coli succinate production
Source: Biotechnol Biofuels. 2016 Dec 1;9:262. doi: 10.1186/s13068-016-0675-y (PMC5134279; doi:10.1186/s13068-016-0675-y)
Supplement: Supplementary file 3 — Additional file 3. Production of succinate by engineering individual PPP enzymes . [file 13068_2016_675_MOESM3_ESM.doc]

**Additional Table S3. Production of succinate by engineering individual PPP enzymes**

| Strain^a^ | Cell mass (g/L)^b^ | Glu concn used  (mM) | Suc concn  (mM) | Suc  yield (mol/mol) | Individual enzymatic activity (U/mg) | Other fermentation product concn (mM) | | |  |
| --- | --- | --- | --- | --- | --- | --- | --- | --- | --- |
|  |  |  |  |  |  | Pyr | Ace | EtOH | |
| Zwf RBSL | | | | | | | | | |
| Suc-T110 | 1.53 | 235 | 263±2 | 1.12±0.03 | 0.13±0.01 (L) | 3±1 | 90±6 | 8±2 | |
| RBSL1-zwf | 1.21 | 235 | 322±3 | 1.37±0.01 | 0.80±0.02 | 3±1 | 85±3 | 4±1 | |
| RBSL2-zwf | 1.47 | 235 | 338±4 | 1.44±0.02 | 1.50±0.03 (M) | 2±1 | 79±3 | 9±3 | |
| RBSL3-zwf | 1.36 | 235 | 322±3 | 1.37±0.01 | 2.16±0.01 | 4±2 | 76±4 | 1±1 | |
| RBSL4-zwf | 1.38 | 235 | 320±4 | 1.36±0.02 | 2.47±0.03 (H) | 4±1 | 84±5 | 3±2 | |
| Pgl RBSL | | | | | | | | | |
| Suc-T110 | 1.56 | 241 | 270±5 | 1.12±0.03 | 0.71±0.06 (L) | 3±1 | 89±5 | 2±1 | |
| RBSL1-pgl | 1.93 | 241 | 321±3 | 1.33±0.01 | 2.44±0.05 | 2±1 | 69±4 | 3±2 | |
| RBSL2-pgl | 1.68 | 241 | 316±4 | 1.31±0.02 | 3.05±0.03 (M) | 1±1 | 63±4 | 3±1 | |
| RBSL3-pgl | 1.57 | 241 | 308±3 | 1.28±0.01 | 5.34±0.09 | 1±1 | 69±5 | 1±1 | |
| RBSL4-pgl | 1.87 | 233 | 294±4 | 1.26±0.02 | 5.74±0.11 (H) | 1±1 | 75±7 | 7±3 | |
| Gnd RBSL | | | | | | | | | |
| Suc-T110 | 1.50 | 244 | 273±4 | 1.12±0.02 | 0.42±0.03 (L) | 3±1 | 93±4 | 8±2 | |
| RBSL1-gnd | 1.46 | 244 | 309±5 | 1.27±0.02 | 2.41±0.06 | 2±1 | 62±5 | 2±1 | |
| RBSL2-gnd | 1.39 | 244 | 314±3 | 1.29±0.01 | 4.90±0.10 | 1±1 | 80±7 | 1±1 | |
| RBSL3-gnd | 1.75 | 244 | 320±2 | 1.31±0.01 | 5.71±0.16 (M) | 1±1 | 78±6 | 3±2 | |
| RBSL4-gnd | 1.16 | 224 | 278±5 | 1.24±0.02 | 11.3±0.23 (H) | 2±1 | 82±9 | 8±4 | |
| rpiA RBSL | | | | | | | | | |
| Suc-T110 | 1.55 | 244 | 273±8 | 1.12±0.03 | 0.19±0.03 (L) | 3±2 | 93±4 | 8±2 | |
| RBSL1-rpiA | 1.48 | 244 | 320±6 | 1.31±0.01 | 0.10±0.01 | 1±1 | 81±5 | 1±1 | |
| RBSL2-rpiA | 1.30 | 224±4 | 280±5 | 1.25±0.02 | 0.15±0.01 | 2±1 | 99±8 | 9±3 | |
| RBSL3-rpiA | 0.56 | 136±3 | 152±3 | 1.12±0.02 | 0.27±0.03 (M) | 3±1 | 77±5 | 14±5 | |
| RBSL4-rpiA | 0.23 | 65±4 | 73±1 | 1.13±0.01 | 0.41±0.04 (H) | 4±2 | 41±4 | 7±3 | |
| Rpe RBSL | | | | | | | | | |
| Suc-T110 | 1.55 | 244 | 273±8 | 1.12±0.03 | 0.27±0.04 (L) | 3±2 | 93±4 | 8±2 | |
| RBSL1-rpe | 1.22 | 201±3 | 232±3 | 1.15±0.01 | 0.54±0.03 | 3±1 | 74±6 | 6±2 | |
| RBSL2-rpe | 0.98 | 168±2 | 203±2 | 1.21±0.01 | 0.79±0.06 (M) | 5±2 | 71±5 | 6±1 | |
| RBSL3-rpe | 0.89 | 157±3 | 182±3 | 1.16±0.02 | 1.14±0.08 | 5±1 | 87±9 | 10±3 | |
| RBSL4-rpe | 0.71 | 119±3 | 137±5 | 1.15±0.04 | 1.36±0.11 (H) | 1±1 | 46±4 | 5±2 | |
| TktA RBSL | | | | | | | | | |
| Suc-T110 | 1.46 | 238 | 267±3 | 1.12±0.01 | 0.07±0.02 (L) | 3±2 | 91±4 | 8±2 | |
| RBSL1-tktA | 1.43 | 238 | 326±2 | 1.37±0.01 | 0.61±0.01 (M) | 2±1 | 60±5 | 7±1 | |
| RBSL2-tktA | 1.39 | 238 | 324±3 | 1.36±0.01 | 0.68±0.02 | 2±1 | 61±4 | 7±2 | |
| RBSL3-tktA | 1.57 | 238 | 319±2 | 1.34±0.01 | 1.10±0.05 (H) | 1±1 | 62±6 | 7±1 | |
| M1-37-tktA | 1.36 | 238 | 300±5 | 1.26±0.02 | 1.20±0.07 | 2±1 | 77±6 | 2±1 | |
| TalB RBSL | | | | | | | | | |
| Suc-T110 | 1.46 | 238 | 267±3 | 1.12±0.01 | 0.054±0.006 (L) | 3±2 | 91±4 | 8±2 | |
| RBSL1-talB | 1.46 | 238 | 324±3 | 1.36±0.01 | 0.14±0.02 | 3±1 | 68±7 | 2±1 | |
| RBSL2-talB | 1.40 | 238 | 333±5 | 1.40±0.01 | 0.20±0.03 (M) | 3±1 | 63±8 | 1±1 | |
| RBSL3-talB | 1.55 | 238 | 321±3 | 1.35±0.01 | 0.23±0.01 | 5±2 | 55±4 | 5±2 | |
| RBSL4-talB | 1.54 | 238 | 309±4 | 1.30±0.02 | 0.26±0.03 (H) | 3±1 | 75±7 | 2±1 | |

^a^ Fermentation was performed in NBS mineral salts medium containing about 5% (wt/v) glucose and 100 mM potassium bicarbonate (37ºC, pH 7.0, 150 rpm, 96 hours).

^b^ Cell mass was calculated from the highest OD_550_ value through the fermentation (1 OD_550_=0.333 g DCW l^-1^).

Abbreviations: Glu, Glucose; Suc, Succinate; Pyr, pyruvate; Ace, acetate; EtOH, ethanol. L, low activity; M, medium activity; H, high activity.
