# Supplementary material for: Systematic engineering of pentose phosphate pathway improves Escherichia coli succinate production
Source: Biotechnol Biofuels. 2016 Dec 1;9:262. doi: 10.1186/s13068-016-0675-y (PMC5134279; doi:10.1186/s13068-016-0675-y)
Supplement: Supplementary file 4 — Additional file 4. Modular engineering PPP for succinate production. [file 13068_2016_675_MOESM4_ESM.doc]

**Additional Table S4. Modular engineering PPP for succinate production**

| Strain^a^ | Cell mass (g/L)^b^ | Glu concn used  (mM) | Suc concn  (mM) | Suc yield (mol/mol) | Other fermentation product concn (mM) | | |
| --- | --- | --- | --- | --- | --- | --- | --- |
|  |  |  |  |  | Pyr | Ace | EtOH |
| Engineering of ZPG module | | | | | | | |
| L-Zwf/L-Pgl/L-Gnd | 1.56 | 252 | 282±5 | 1.12±0.02 | 2±1 | 90±8 | 6±1 |
| L-Zwf/M-Pgl/M-Gnd | 2.18 | 252 | 313±4 | 1.24±0.02 | 4±1 | 68±4 | 4±1 |
| M-Zwf/M-Pgl/M-Gnd | 1.68 | 245 | 353±2 | 1.44±0.03 | 0 | 70±5 | 0 |
| H-Zwf/M-Pgl/M-Gnd | 1.53 | 245 | 373±2 | 1.52±0.02 | 0 | 64±8 | 0 |
| L-Zwf /H-Pgl/H-Gnd | 1.40 | 198±4 | 230±3 | 1.16±0.02 | 2±1 | 63±3 | 9±2 |
| M-Zwf/H-Pgl/H-Gnd | 0.37 | 49±5 | 53±4 | 1.05±0.08 | 0 | 25±3 | 0 |
| H-Zwf/H-Pgl/H-Gnd | 0.23 | 49±6 | 34±1 | 0.69±0.02 | 0 | 17±1 | 0 |
| Engineering of RR module | | | | | | | |
| L-Rpe/L-Rpi | 1.40 | 240 | 271±2 | 1.12±0.02 | 1±0 | 85±3 | 4±1 |
| L-Rpe/M-Rpi | 0.26 | 65±4 | 73±1 | 1.13±0.01 | 4±2 | 41±4 | 7±3 |
| M-Rpe/L-Rpi | 0.84 | 136±2 | 169±3 | 1.24±0.02 | 2±1 | 45±2 | 4±1 |
| M-Rpe/M-Rpi | 0.60 | 87±1 | 104±2 | 1.20±0.02 | 1±0 | 48±3 | 4±1 |
| H-Rpe/L-Rpi | 0.67 | 109±3 | 119±3 | 1.09±0.03 | 1±1 | 61±4 | 6±2 |
| H-Rpe/M-Rpi | 0.66 | 100±3 | 108±5 | 1.08±0.05 | 1±0 | 57±3 | 5±1 |
| H-Rpe/H-Rpi | 0.63 | 96±4 | 106±4 | 1.10±0.04 | 1±1 | 51±4 | 11±2 |
| Engineering of TT module | | | | | | | |
| L-Tkt/L-Tal | 1.50 | 238 | 268±3 | 1.12±0.02 | 2±1 | 80±5 | 1±1 |
| M-Tkt/L-Tal | 1.43 | 238 | 325±2 | 1.37±0.01 | 2±1 | 58±5 | 7±1 |
| M-Tkt/M-Tal | 1.50 | 238 | 328±1 | 1.38±0.01 | 3±1 | 71±5 | 4±1 |
| M-Tkt/H-Tal | 1.40 | 238 | 336±4 | 1.41±0.02 | 5±1 | 61±4 | 3±1 |
| H-Tkt/L-Tal | 1.57 | 238 | 320±2 | 1.34±0.01 | 1±1 | 62±6 | 7±1 |
| H-Tkt/M-Tal | 1.52 | 238 | 332±2 | 1.39±0.01 | 4±1 | 70±3 | 5±1 |
| H-Tkt/H-Tal | 1.59 | 225±3 | 296±2 | 1.32±0.01 | 4±1 | 81±6 | 7±3 |
| Combinational utilization of PPP modules and SthA | | | | | | | |
| Suc-T110 | 1.51 | 247 | 277±2 | 1.12±0.02 | 3±1 | 92±6 | 9±2 |
| +TT | 1.40 | 245±2 | 346±4 | 1.41±0.02 | 5±1 | 80±4 | 3±1 |
| +ZPG | 1.69 | 247 | 376±15 | 1.52±0.02 | 0 | 76 | 0 |
| +ZPG/+TT  (Suc-P01) | 1.73 | 244±6 | 373±16 | 1.54±0.02 | 0 | 70 | 0 |
| +ZPG/+TT/+SthA (Suc-P02) | 1.71 | 239±8 | 382±10 | 1.61±0.04 | 0 | 56 | 0 |

^a^ Fermentation was performed in NBS mineral salts medium containing about 5% (wt/v) glucose and 100 mM potassium bicarbonate (37ºC, pH 7.0, 150 rpm, 96 hours).

^b^ Cell mass was calculated from the highest OD_550_ value through the fermentation (1 OD_550_=0.333 g DCW l^-1^).

Abbreviations: Glu, Glucose; Suc, Succinate; Pyr, pyruvate; Ace, acetate; EtOH, ethanol. ZPG, Zwf/Pgl/Gnd module; RR, Rpi/Rpe module; TT, Tkt/Tal module; L, low activity; M, medium activity; H, high activity.
