# Supplementary material for: Systematic engineering of pentose phosphate pathway improves Escherichia coli succinate production
Source: Biotechnol Biofuels. 2016 Dec 1;9:262. doi: 10.1186/s13068-016-0675-y (PMC5134279; doi:10.1186/s13068-016-0675-y)
Supplement: Supplementary file 5 — Additional file 5. Solving metabolic burdens through multivariate modular engineering. [file 13068_2016_675_MOESM5_ESM.doc]

**Additional Table S5. Solving metabolic burdens through multivariate modular engineering**

| Genetic modifications^a^ | Cell mass (g/L)^b^ | Glu concn used  (mM) | Suc concn  (mM) | Suc yield (mol/mol) | Other fermentation product concn (mM) | | |
| --- | --- | --- | --- | --- | --- | --- | --- |
|  |  |  |  |  | Pyr | Ace | EtOH |
| Under H-ZPG | | | | | | | |
| L-RR/L-TT | 0.23 | 49±6 | 34±1 | 0.69±0.02 | 2±1 | 17±1 | 1±1 |
| L-RR/M-TT | 1.04 | 91±2 | 94±2 | 1.03±0.02 | 3±1 | 33±2 | 3±1 |
| L-RR/H-TT | 1.12 | 87±2 | 84±2 | 0.96±0.02 | 3±1 | 46±3 | 1±1 |
| M-RR/L-TT | 0.49 | 38±3 | 43±3 | 1.14±0.08 | 1±1 | 34±3 | 13±4 |
| M-RR/M-TT | 1.63 | 203±5 | 237±6 | 1.17±0.03 | 2±1 | 80±3 | 5±2 |
| M-RR/H-TT | 1.17 | 134±4 | 151±8 | 1.12±0.06 | 2±1 | 55±6 | 6±2 |
| H-RR/L-TT | 0.65 | 60±1 | 66±2 | 1.10±0.03 | 1±1 | 35±3 | 6±1 |
| H-RR/M-TT | 0.28 | 31±2 | 20±2 | 0.63±0.07 | 1±1 | 18±2 | 9±1 |
| H-RR/H-TT | 0.29 | 34±3 | 27±2 | 0.79±0.06 | 1±0 | 20±2 | 6±1 |
| Under H-RR | | | | | | | |
| L-ZPG/L-TT | 0.63 | 96±4 | 106±4 | 1.10±0.04 | 1±1 | 51±4 | 11±2 |
| L-ZPG/M-TT | 0.58 | 91±2 | 91±4 | 1.00±0.04 | 1±1 | 50±2 | 6±2 |
| L-ZPG/H-TT | 0.66 | 102±2 | 109±3 | 1.07±0.03 | 2±1 | 51±3 | 12±4 |
| M-ZPG/L-TT | 1.11 | 213±2 | 263±5 | 1.23±0.02 | 3±1 | 85±6 | 7±2 |
| M-ZPG/M-TT | 1.93 | 250 | 314±4 | 1.26±0.02 | 1±0 | 63±3 | 1±1 |
| M-ZPG/H-TT | 1.72 | 250 | 321±4 | 1.28±0.02 | 1±1 | 55±2 | 3±1 |
| H-ZPG/L-TT | 0.65 | 60±1 | 66±2 | 1.10±0.03 | 1±1 | 35±3 | 6±1 |
| H-ZPG/M-TT | 0.28 | 31±2 | 20±2 | 0.63±0.07 | 1±1 | 18±2 | 9±1 |
| H-ZPG/H-TT | 0.29 | 34±3 | 27±2 | 0.79±0.06 | 1±0 | 20±2 | 6±1 |
| Under H-TT | | | | | | | |
| L-ZPG/L-RR | 1.59 | 225±1 | 296±2 | 1.32±0.01 | 4±1 | 81±6 | 7±3 |
| L-ZPG/M-RR | 1.89 | 213±3 | 302±2 | 1.41±0.01 | 2±1 | 63±6 | 6±3 |
| L-ZPG/H-RR | 0.66 | 102±2 | 109±3 | 1.07±0.03 | 2±1 | 51±3 | 12±4 |
| M-ZPG/L-RR | 1.95 | 250 | 323±4 | 1.29±0.02 | 2±1 | 47±2 | 3±1 |
| M-ZPG/M-RR | 2.00 | 248 | 328±4 | 1.32±0.02 | 1±0 | 53±4 | 2±1 |
| M-ZPG/H-RR | 1.72 | 250 | 321±4 | 1.28±0.02 | 1±1 | 55±2 | 3±1 |
| H-ZPG/L-RR | 1.12 | 87±2 | 84±2 | 0.96±0.02 | 3±1 | 46±3 | 1±1 |
| H-ZPG/M-RR | 1.17 | 134±4 | 151±8 | 1.12±0.06 | 2±1 | 55±6 | 6±2 |
| H-ZPG/H-RR | 0.29 | 34±3 | 27±2 | 0.79±0.06 | 1±0 | 20±2 | 6±1 |

^a^ Fermentation was performed in NBS mineral salts medium containing about 5% (wt/v) glucose and 100 mM potassium bicarbonate (37ºC, pH 7.0, 150 rpm, 96 hours).

^b^ Cell mass was calculated from the highest OD_550_ value through the fermentation (1 OD_550_=0.333 g DCW l^-1^).

^c^ Abbreviations: Glu, Glucose; Suc, Succinate; Pyr, pyruvate; Ace, acetate; EtOH, ethanol. ZPG, Zwf/Pgl/Gnd module; RR, Rpi/Rpe module; TT, Tkt/Tal module; L, low activity; M, medium activity; H, high activity.
