# Supplementary material for: Systematic engineering of pentose phosphate pathway improves Escherichia coli succinate production
Source: Biotechnol Biofuels. 2016 Dec 1;9:262. doi: 10.1186/s13068-016-0675-y (PMC5134279; doi:10.1186/s13068-016-0675-y)
Supplement: Supplementary file 6 — Additional file 6. Genotypes of all strains used in this study. [file 13068_2016_675_MOESM6_ESM.doc]

**Additional Table S6. Genotypes of all strains used in this study**

| Strain | Genotype |
| --- | --- |
| Suc-T110 | *E. coli* ATCC 8739 ∆*pflB* ∆*ldhA* ∆*ptsI* *Ppck*-galP pck** |
| RBSL1-zwf | Suc-T110, RBSL1-*zwf* |
| RBSL2-zwf | Suc-T110, RBSL2-*zwf* (M-Zwf) |
| RBSL3-zwf | Suc-T110, RBSL3-*zwf* |
| RBSL4-zwf | Suc-T110, RBSL4-*zwf* (H-Zwf) |
| RBSL1-pgl | Suc-T110, RBSL1-*pgl* |
| RBSL2-pgl | Suc-T110, RBSL2-*pgl* (M-Pgl) |
| RBSL3-pgl | Suc-T110, RBSL3-*pgl* |
| RBSL4-pgl | Suc-T110, RBSL4-*pgl* (H-Pgl) |
| RBSL1-gnd | Suc-T110, RBSL1-*gnd* |
| RBSL2-gnd | Suc-T110, RBSL2-*gnd* |
| RBSL3-gnd | Suc-T110, RBSL3-*gnd* (M-Gnd) |
| RBSL4-gnd | Suc-T110, RBSL4-*gnd* (H-Gnd) |
| RBSL1-rpiA | Suc-T110, RBSL1-*rpiA* |
| RBSL2-rpiA | Suc-T110, RBSL2-*rpiA* |
| RBSL3-rpiA | Suc-T110, RBSL3-*rpiA* (M-Rpi) |
| RBSL4-rpiA | Suc-T110, RBSL4-*rpiA* (H-Rpi) |
| RBSL1-rpe | Suc-T110, RBSL1-*rpe* |
| RBSL2-rpe | Suc-T110, RBSL2-*rpe* (M-Rpe) |
| RBSL3-rpe | Suc-T110, RBSL3-*rpe* |
| RBSL4-rpe | Suc-T110, RBSL4-*rpe* (H-Rpe) |
| RBSL1-tktA | Suc-T110, RBSL1-*tktA* (M-Tkt) |
| RBSL2-tktA | Suc-T110, RBSL2-*tktA* |
| RBSL3-tktA | Suc-T110, RBSL3-*tktA* (H-Tkt) |
| RBSL4-tktA | Suc-T110, RBSL4-*tktA* |
| RBSL1-talB | Suc-T110, RBSL1-*talB* |
| RBSL2-talB | Suc-T110, RBSL2-*talB* (M-Tal) |
| RBSL3-talB | Suc-T110, RBSL3-*talB* |
| RBSL4-talB | Suc-T110, RBSL4-*talB* (H-Tal) |
| L-Zwf/L-Pgl/L-Gnd | Suc-T110 |
| L-Zwf/M-Pgl/M-Gnd | Suc-T110, RBSL2-*pgl*, RBSL3-*gnd* |
| M-Zwf/M-Pgl/M-Gnd | Suc-T110, RBSL2-*zwf*, RBSL2-*pgl*, RBSL3-*gnd* |
| H-Zwf/M-Pgl/M-Gnd | Suc-T110, RBSL4-*zwf*, RBSL2-*pgl*, RBSL3-*gnd* |
| L-Zwf /H-Pgl/H-Gnd | Suc-T110, RBSL4-*pgl*, RBSL4-*gnd* |
| M-Zwf/H-Pgl/H-Gnd | Suc-T110, RBSL2-*zwf*, RBSL4-*pgl*, RBSL4-*gnd* |
| H-Zwf/H-Pgl/H-Gnd | Suc-T110, RBSL4-*zwf*, RBSL4-*pgl*, RBSL4-*gnd* |
| L-Rpe/L-Rpi | Suc-T110 |
| L-Rpe/M-Rpi | Suc-T110, RBSL3-*rpiA* |
| M-Rpe/L-Rpi | Suc-T110, RBSL2-*rpe* |
| M-Rpe/M-Rpi | Suc-T110, RBSL3-*rpiA*, RBSL2-*rpe* |
| H-Rpe/L-Rpi | Suc-T110, RBSL4-*rpe* |
| H-Rpe/H-Rpi | Suc-T110, RBSL4-*rpiA*, RBSL4-*rpe* |
| L-Tkt/L-Tal | Suc-T110 |
| M-Tkt/L-Tal | Suc-T110, RBSL1-*tktA* |
| M-Tkt/M-Tal | Suc-T110, RBSL1-*tktA*, RBSL2-*talB* |
| M-Tkt/H-Tal | Suc-T110, RBSL1-*tktA*, RBSL4-*talB* |
| H-Tkt/L-Tal | Suc-T110, RBSL3-*tktA* |
| H-Tkt/M-Tal | Suc-T110, RBSL3-*tktA*, RBSL2-*talB* |
| H-Tkt/H-Tal | Suc-T110, RBSL3-*tktA*, RBSL4-*talB* |
| +TT | Suc-T110, RBSL1-*tktA*, RBSL4-*talB* |
| +ZPG | Suc-T110, RBSL4-*zwf*, RBSL2-*pgl*, RBSL3-*gnd* |
| +ZPG/+TT  (Suc-P01) | Suc-T110, RBSL4-*zwf*, RBSL2-*pgl*, RBSL3-*gnd*, RBSL1-*tktA*, RBSL4-*talB* |
| +ZPG/+TT/+SthA (Suc-P02) | Suc-T110, RBSL4-*zwf*, RBSL2-*pgl*, RBSL3-*gnd*, RBSL1-*tktA*, RBSL4-*talB,* RBS3-*sthA* |
| H-ZPG/L-RR/L-TT | Suc-T110, RBSL4-*zwf*, RBSL4-*pgl*, RBSL4-*gnd* |
| H-ZPG/L-RR/M-TT | Suc-T110, RBSL4-*zwf*, RBSL4-*pgl*, RBSL4-*gnd*, RBSL1-*tktA*, RBSL2-*talB* |
| H-ZPG/L-RR/H-TT | Suc-T110, RBSL4-*zwf*, RBSL4-*pgl*, RBSL4-*gnd*, RBSL4-*tktA*, RBSL4-*talB* |
| H-ZPG/M-RR/L-TT | Suc-T110, RBSL4-*zwf*, RBSL4-*pgl*, RBSL4-*gnd*, RBSL3-*rpiA*, RBSL2-*rpe* |
| H-ZPG/M-RR/M-TT | Suc-T110, RBSL4-*zwf*, RBSL4-*pgl*, RBSL4-*gnd*, RBSL3-*rpiA*, RBSL2-*rpe*, RBSL1-*tktA*, RBSL2-*talB* |
| H-ZPG/M-RR/H-TT | Suc-T110, RBSL4-*zwf*, RBSL4-*pgl*, RBSL4-*gnd*, RBSL3-*rpiA*, RBSL2-*rpe*, RBSL3-*tktA*, RBSL4-*talB* |
| H-ZPG/H-RR/L-TT | Suc-T110, RBSL4-*zwf*, RBSL4-*pgl*, RBSL4-*gnd*, RBSL4-*rpiA*, RBSL4-*rpe* |
| H-ZPG/H-RR/M-TT | Suc-T110, RBSL4-*zwf*, RBSL4-*pgl*, RBSL4-*gnd*, RBSL4-*rpiA*, RBSL4-*rpe*, RBSL1-*tktA*, RBSL2-*talB* |
| H-ZPG/H-RR/H-TT | Suc-T110, RBSL4-*zwf*, RBSL4-*pgl*, RBSL4-*gnd*, RBSL4-*rpiA*, RBSL4-*rpe*, RBSL3-*tktA*, RBSL4-*talB* |
| L-ZPG/H-RR/L-TT | Suc-T110, RBSL4-*rpiA*, RBSL4-*rpe* |
| L-ZPG/H-RR/M-TT | Suc-T110, RBSL4-*rpiA*, RBSL4-*rpe*, RBSL1-*tktA*, RBSL2-*talB* |
| L-ZPG/H-RR/H-TT | Suc-T110, RBSL4-*rpiA*, RBSL4-*rpe*, RBSL3-*tktA*, RBSL4-*talB* |
| M-ZPG/H-RR/L-TT | Suc-T110, RBSL2-*zwf*, RBSL2-*pgl*, RBSL3-*gnd*, RBSL4-*rpiA*, RBSL4-*rpe*, |
| M-ZPG/H-RR/M-TT | Suc-T110, RBSL2-*zwf*, RBSL2-*pgl*, RBSL3-*gnd*, RBSL4-*rpiA*, RBSL4-*rpe*, RBSL1-*tktA*, RBSL2-*talB* |
| M-ZPG/H-RR/H-TT | Suc-T110, RBSL2-*zwf*, RBSL2-*pgl*, RBSL3-*gnd*, RBSL4-*rpiA*, RBSL4-*rpe*, RBSL3-*tktA*, RBSL4-*talB* |
| H-ZPG/H-RR/L-TT | Suc-T110, RBSL4-*zwf*, RBSL4-*pgl*, RBSL4-*gnd*, RBSL4-*rpiA*, RBSL4-*rpe* |
| H-ZPG/H-RR/M-TT | Suc-T110, RBSL4-*zwf*, RBSL4-*pgl*, RBSL4-*gnd*, RBSL4-*rpiA*, RBSL4-*rpe*, RBSL1-*tktA*, RBSL2-*talB* |
| H-ZPG/H-RR/H-TT | Suc-T110, RBSL4-*zwf*, RBSL4-*pgl*, RBSL4-*gnd*, RBSL4-*rpiA*, RBSL4-*rpe*, RBSL3-*tktA*, RBSL4-*talB* |
| L-ZPG/L-RR/H-TT | Suc-T110, RBSL3-*tktA*, RBSL4-*talB* |
| L-ZPG/M-RR/H-TT | Suc-T110, RBSL3-*rpiA*, RBSL2-*rpe*, RBSL3-*tktA*, RBSL4-*talB* |
| L-ZPG/H-RR/H-TT | Suc-T110, RBSL4-*rpiA*, RBSL4-*rpe*, RBSL3-*tktA*, RBSL4-*talB* |
| M-ZPG/L-RR/H-TT | Suc-T110, RBSL2-*zwf*, RBSL2-*pgl*, RBSL3-*gnd*, RBSL3-*tktA*, RBSL4-*talB* |
| M-ZPG/M-RR/H-TT | Suc-T110, RBSL2-*zwf*, RBSL2-*pgl*, RBSL3-*gnd*, RBSL3-*rpiA*, RBSL2-*rpe*, RBSL3-*tktA*, RBSL4-*talB* |
| M-ZPG/H-RR/H-TT | Suc-T110, RBSL2-*zwf*, RBSL2-*pgl*, RBSL3-*gnd*, RBSL4-*rpiA*, RBSL4-*rpe*, RBSL3-*tktA*, RBSL4-*talB* |
| H-ZPG/L-RR/H-TT | Suc-T110, RBSL4-*zwf*, RBSL4-*pgl*, RBSL4-*gnd*, RBSL3-*tktA*, RBSL4-*talB* |
| H-ZPG/M-RR/H-TT | Suc-T110, RBSL4-*zwf*, RBSL4-*pgl*, RBSL4-*gnd*, RBSL3-*rpiA*, RBSL2-*rpe*, RBSL3-*tktA*, RBSL4-*talB* |
| H-ZPG/H-RR/H-TT | Suc-T110, RBSL4-*zwf*, RBSL4-*pgl*, RBSL4-*gnd*, RBSL4-*rpiA*, RBSL4-*rpe*, RBSL3-*tktA*, RBSL4-*talB* |
