# Supplementary material for: Systematic engineering of pentose phosphate pathway improves Escherichia coli succinate production
Source: Biotechnol Biofuels. 2016 Dec 1;9:262. doi: 10.1186/s13068-016-0675-y (PMC5134279; doi:10.1186/s13068-016-0675-y)
Supplement: Supplementary file 7 — Additional file 7. Primers used in this study. [file 13068_2016_675_MOESM7_ESM.doc]

**Additional Table S7.** Primers used in this study

| **Primer** | **Sequence** |
| --- | --- |
| **Construction of *zwf* RBSL** | |
| zwf-cat-sacB-up | ATCAGTTTTGCCGCACTTTGCGCGCTTTTCCCGTAATCGCACGGGTGGATAAGTGTGACGGAAGATCACTTCGCA |
| zwf-cat-sacB-down | CCAGGGTATACTTGTAATTTTCTTACGGTGCACTGTACTGCTTTTACGAGCTTGTTATTTGTTAACTGTTAATTGTCCT |
| zwf-P-up | ATCAGTTTTGCCGCACTTTGCGCGCTTTTCCCGTAATCGCACGGGTGGATAAGTTATCTCTGGCGGTGTTGAC |
| zwf-RBSL-down | GCGCCGAAAATGACCAGGTCACAGGCCTGGGCTGTTTGCGTTACCGCCAT*NNNNNNY*CTCCTGGTTTAAACGTACATG |
| zwf-EX-up | CATGGCAAAGTAGTTAATGG |
| zwf-EX-down | GACTCACGGGTAATGACGAT |
| **Construction of *pgl* RBSL** | |
| pgl-cat-sacB-up | TTCAGCATTCACCGCCAAAAGCGACTAATTTTAGCTGTTACAGTCAGTTGGCGTTGGCCGATTCATTA |
| pgl-cat-sacB-down | ACGTGAATTTGCTGGCTCTCAGGGCTGGCGATATAAACTGTTTGCTTCATGGAGAAAATACCGCATCAGG |
| pgl-P-up | TTCAGCATTCACCGCCAAAAGCGACTAATTTTAGCTGTTACAGTCAGTTGTTATCTCTGGCGGTGTTGAC |
| pgl-RBSL-down | ACGTGAATTTGCTGGCTCTCAGGGCTGGCGATATAAACTGTTTGCTTCAT*NNNNNNY*CTCCTGGTTTAAACGTACATG |
| pgl-EX-up | GTGATGGCGACCTGTGACGA |
| pgl-EX-down | GGGCGAACACCAACATAGAG |
| **Construction of *gnd* RBSL** | |
| gnd-cat-sacB-up | CTTACTAATTTAATGAATAGAACTCAATTGTATGTCCATTTGATTCAGTCGCGTTGGCCGATTCATTA |
| gnd-cat-sacB-down | TTGCGCCCCATCACTGCCATACCGACTACGCCGATCTGTTGCTTTGACATGGAGAAAATACCGCATCAGG |
| gnd-P-up | CTTACTAATTTAATGAATAGAACTCAATTGTATGTCCATTTGATTCAGTCTTATCTCTGGCGGTGTTGAC |
| gnd-RBSL-down | TTGCGTCCCATCACTGCCATACCGACTACGCCGATCTGTTGCTTGGACAT*NNNNNNY*CTCCTGGTTTAAACGTACATG |
| gnd-EX-up | GGTCCTTGCTATAAGAGTGA |
| gnd-EX-down | ACGGTTACGACGGATGGTGT |
| **Construction of *rpiA* RBSL** | |
| rpiA-cat-sacB-up | CGCGGCTTGCCAACGGGGTCTGAATCGCTTTTTTTGTATATAATGCGTGTGCGTTGGCCGATTCATTA |
| rpiA-cat-sacB-down | TTTTCAATTCATCCTGCGTCATGATCGTTTCGCCTGTGGTATGAAATTTCGGAGAAAATACCGCATCAGG |
| rpiA-P-up | CGCGGCTTGCCAACGGGGTCTGAATCGCTTTTTTTGTATATAATGCGTGTTTATCTCTGGCGGTGTTGAC |
| rpiA-RBSL-down | TACTGAAGTGCCGCCCATCCTACTGCTTTTTTCAATTCATCCTGCGTCAT*NNNNNNY*CTCCTGGTTTAAACGTACATG |
| rpiA-EX-up | AACCTGCTTGGAAGCGTCTG |
| rpiA-EX-down | TGTTGAATGGCGTGGCGTTA |
| **Construction of *rpe* RBSL** | |
| rpe-cat-sacB-up | TCAGCGCGCAAAATTGCATGTCGTCAAAGTTCGACGCAGTATAAGCAGCAGCGTTGGCCGATTCATTA |
| rpe-cat-sacB-down | TGGTTTGTACAAAGCCAGCAGTTCGTCCACCTTTTTACGTGTGCCGCCGTGGAGAAAATACCGCATCAGG |
| rpe-P-up | TCAGCGCGCAAAATTGCATGTCGTCAAAGTTCGACGCAGTATAAGCAGCATTATCTCTGGCGGTGTTGAC |
| rpe-RBSL-down | CGGGCAAAATCAGCCGACAGAATTGAGGGGGCAATCAAATACTGTTTCAT*NNNNNNY*CTCCTGGTTTAAACGTACATG |
| rpe-EX-up | GACCCGCTTTACAGCCATTT |
| rpe-EX-down | TTGTGAGTCTTACGCCGATA |
| **Construction of *tktA* RBSL** | |
| tktA-cat-sacB-up | AAATGCGCCGTTTGCAGGTGAATCGACGCTCAGTCTCAGTATAAGGAATGTGACGGAAGATCACTTCGCA |
| tktA-cat-sacB-down | TCCATGCTCAGCGCACGAATAGCATTGGCAAGCTCTTTACGTGAGGACATTTATTTGTTAACTGTTAATTGTCCT |
| tktA-P-up | AAATGCGCCGTTTGCAGGTGAATCGACGCTCAGTCTCAGTATAAGGAATTATCTCTGGCGGTGTTGAC |
| tktA-RBSL-down | TCCATGCTCAGCGCACGAATAGCATTGGCAAGCTCTTTACGTGAGGACAT*NNNNNNY*CTCCTGGTTTAAACGTACATG |
| tktA-EX-up | TCAGGAAATCACGCCACA |
| tktA-EX-down | ATCCGTCATCATATCCATCA |
| **Construction of *talB* RBSL** | |
| talB-cat-sacB-up | AGTCTCGCCTGGCGATAACCGTCTTGTCGGCGGTTGCGCTGACGTTGCGTCGTGTGTGACGGAAGATCACTTCGCA |
| talB-cat-sacB-down | TCATGATAGTATTTCTCTTTAAACAGCTTGTTAGGGGGATGTAACCGGTCTGCTTATTTGTTAACTGTTAATTGTCCT |
| talB-P-up | AGTCTCGCCTGGCGATAACCGTCTTGTCGGCGGTTGCGCTGACGTTGCGTCGTGTTATCTCTGGCGGTGTTGAC |
| talB-RBSL-down | TCGGCCACTACGGTGGTGTACTGACGAAGGGAGGTCAATTTGTCCGTCAT*NNNNNNY*CTCCTGGTTTAAACGTACATG |
| talB-EX-up | CCGAAGAGCAGGTAAATCAT |
| talB-EX-down | TACCAGCATCGTTGTAGAGT |
