# Supplementary material for: Systematic engineering of pentose phosphate pathway improves Escherichia coli succinate production
Source: Biotechnol Biofuels. 2016 Dec 1;9:262. doi: 10.1186/s13068-016-0675-y (PMC5134279; doi:10.1186/s13068-016-0675-y)
Supplement: Supplementary file 8 — Additional file 8. Kinetic parameters of E. coli PPP enzymes. [file 13068_2016_675_MOESM8_ESM.doc]

**Additional Table S8. Kinetic parameters of *E. coli*** PPP enzymes

| Enzyme | EC number | Function | Substrate | Kcat (S^-1^) | Km (mM) | Kcat/Km (1/mM S^-1^) | Reference |
| --- | --- | --- | --- | --- | --- | --- | --- |
| Zwf | 1.1.1.49 | Glucose-6-phosphate dehydrogenase | Glucose 6-phosphate | 165 | 0.0163 | 10122 |  |
|  |  |  | NADP^+^ | 174 | 0.0053 | 23200 |  |
| Pgl | 3.1.1.31 | 6-phosphogluconolactonase | 6-phosphoglucono-δ-lactone | NR | NR | NR |  |
| Gnd | 1.1.1.44 | Phosphogluconate dehydrogenase | 6-phospho-gluconate | 10.2 | 0.093 | 110 |  |
|  |  |  | NADP^+^ | 21.1 | 0.049 | 430 |  |
| RpiA | 5.3.1.6 | Ribosephosphate isomerase A | Ribose 5-phosphate | 2100 | 3.1 | 680 |  |
| Rpe | 5.1.3.1 | Ribulose-5-phosphate 3-epimerase | Ribulose-5-phosphate | NR | NR | NR |  |
| Tkt | 2.2.1.1 | Transketolase | Xylulose-5-phosphate | NR | 0.16 | NR |  |
|  |  |  | Ribose 5-phosphate | NR | 1.4 | NR |  |
|  |  |  | Erythrose-4-phosphate | NR | 0.09 | NR |  |
| Tal | 2.2.1.2 | Transaldolase | Sedoheptulose-7-phosphate | NR | 0.285 | NR |  |
|  |  |  | Glyceraldehyde 3-phosphate | 13 | 1.9 | 6.6 |  |

NR, not recorded.

**References**

1. Olavarria K, Valdes D, Cabrera R. The cofactor preference of glucose-6-phosphate dehydrogenase from *Escherichia coli-*-modeling the physiological production of reduced cofactors. *FEBS J.* 2012;279:2296-2309.

2. Chen YY, Ko TP, Chen WH, Lo LP, Lin CH, Wang AH. Conformational changes associated with cofactor/substrate binding of 6-phosphogluconate dehydrogenase from *Escherichia coli* and *Klebsiella pneumoniae*: Implications for enzyme mechanism. *J Struct Biol.* 2010;169:25-35.

3. Zhang RG, Andersson CE, Savchenko A, Skarina T, Evdokimova E, Beasley S, Arrowsmith CH, Edwards AM, Joachimiak A, Mowbray SL. Structure of *Escherichia coli* ribose-5-phosphate isomerase: A ubiquitous enzyme of the pentose phosphate pathway and the Calvin cycle. *Structure.* 2003;11:31-42.

4. Schenk G, Duggleby RG, Nixon PF. Properties and functions of the thiamin diphosphate dependent enzyme transketolase. *Int J Biochem Cell Biol.* 1998;30:1297-1318.

5. Sprenger GA, Schorken U, Sprenger G, Sahm H: Transaldolase B of *Escherichia coli* K-12: cloning of its gene, *talB*, and characterization of the enzyme from recombinant strains. *J Bacteriol.* 1995;177:5930-5936.

6. Rale M, Schneider S, Sprenger GA, Samland AK, Fessner WD. Broadening deoxysugar glycodiversity: natural and engineered transaldolases unlock a complementary substrate space. *Chemistry.* 2011;17:2623-2632.
